# Diagnostic Accuracy of Point-of-Care Tests for Hepatitis C Virus Infection: A Systematic Review and Meta-Analysis

**DOI:** 10.1371/journal.pone.0121450

**Published:** 2015-03-27

**Authors:** Mehnaaz Sultan Khuroo, Naira Sultan Khuroo, Mohammad Sultan Khuroo

**Affiliations:** 1 Government Medical College, Srinagar, Kashmir, J&K, India; 2 Digestive Diseases Centre, Dr Khuroo Medical Clinic, Srinagar, Kashmir, J&K, India; The Chinese University of Hong Kong, HONG KONG

## Abstract

**Background:**

Point-of-care tests provide a plausible diagnostic strategy for hepatitis C infection in economically impoverished areas. However, their utility depends upon the overall performance of individual tests.

**Methods:**

A literature search was conducted using the metasearch engine Mettā, a query interface for retrieving articles from five leading medical databases. Studies were included if they employed point-of-care tests to detect antibodies of hepatitis C virus and compared the results with reference tests. Two reviewers performed a quality assessment of the studies and extracted data for estimating test accuracy.

**Findings:**

Thirty studies that had evaluated 30 tests fulfilled the inclusion criteria. The overall pooled sensitivity, specificity, positive likelihood-ratio, negative likelihood-ratio and diagnostic odds ratio for all tests were 97.4% (95% CI: 95.9–98.4), 99.5% (99.2–99.7), 80.17 (55.35–116.14), 0.03 (0.02–0.04), and 3032.85 (1595.86–5763.78), respectively. This suggested a high pooled accuracy for all studies. We found substantial heterogeneity between studies, but none of the subgroups investigated could account for the heterogeneity. Genotype diversity of HCV had no or minimal influence on test performance. Of the seven tests evaluated in the meta-regression model, OraQuick had the highest test sensitivity and specificity and showed better performance than a third generation enzyme immunoassay in seroconversion panels. The next highest test sensitivities and specificities were from TriDot and SDBioline, followed by Genedia and Chembio. The Spot and Multiplo tests produced poor test sensitivities but high test specificities. Nine of the remaining 23 tests produced poor test sensitivities and specificities and/or showed poor performances in seroconversion panels, while 14 tests had high test performances with diagnostic odds ratios ranging from 590.70 to 28822.20.

**Conclusions:**

Performances varied widely among individual point-of-care tests for diagnosis of hepatitis C virus infection. Physicians should consider this while using specific tests in clinical practice.

## Introduction

Hepatitis C is a global health problem [1]. Approximately 2–3% of the world’s population is chronically infected with hepatitis C virus (HCV), which amounts to an estimated 170 million persons. Chronic hepatitis C is associated with significant morbidity and mortality. HCV contributes to 27% of cirrhosis, 25% of hepatocellular carcinoma, and causes more than 350,000 deaths each year [2]. Screening of HCV infection is therefore mandatory in many high-risk epidemiologic settings [3,4]. In addition, testing of blood and blood products is essential for preventing HCV infection of recipients [5]. The use of an enzyme immunoassay (EIA) to detect HCV antibodies (anti-HCV) followed by nucleic acid testing for HCV RNA is standard practice for diagnostic evaluation of HCV infection in developed countries [6,7]. These tests require sophisticated equipment, trained technicians, a continuous supply of electricity, and high facility costs. Hence they are unsuitable for use in regions with limited resources [8].

Since the 1990s, several point-of-care tests have been developed that primarily use serum, plasma, whole blood and oral fluid to test for anti-HCV [9]. Manufacturers claim that these tests have high clinical and analytical sensitivity, so these tests are used widely in many settings in developing countries, including blood banks. However, several vital uncertainties remain regarding their use, including: (i) the accuracy of individual tests, (ii) the comparative efficacy of the different tests, and (iii) how the performances of tests vary based on HCV genotype diversity, HIV/HCV co-infections, HCV performance panels, or other factors [10].

A recent meta-analysis of the accuracy of point-of-care tests for hepatitis C attempted to address some of those uncertainties [11]. However, the study’s relevance was limited for many reasons. (i) A well-conducted, major study of the accuracies of point-of-care tests had been published by then but was not included in the meta-analysis [12]. (ii) The study did not address the issue of inconclusive test results, or how to analyze and report such data. We believe that complete transparency about the handling of inconclusive results in meta-analyses is essential for the reader to understand how key summary statistics have been calculated [13]. (iii) It did not evaluate heterogeneity of the data (i.e., the differences in reported estimates among studies) and its potential causes, an important component of meta-analyses [14]. (iv) The analytical sensitivities of the tests based on seroconversion panels, HCV genotypes, and cross-reactive sera (especially for HIV) were not assessed, which would have affected conclusions about the tests being evaluated. (v) The accuracies of individual tests were not evaluated, which obfuscates the real purpose of the meta-analysis.

We believe that recommendations for the use of point-of-care tests can have far-reaching effects on healthcare in developing countries, so they must be made carefully. For example, recommending tests of low analytical sensitivity for blood banks can pose a serious threat to recipients from infected donors. Accordingly, we conducted a comprehensive systematic review and meta-analysis of studies that assessed the diagnostic accuracy and applicability of point-of-care tests for hepatitis C.

## Materials and Methods

### Protocol

We conducted a systematic review and meta-analysis of studies that evaluated the accuracy of point-of-care tests for HCV. We established a protocol that specified several aspects of the meta-analysis, following the guidelines of PRISMA (Preferred Reporting Items for Systematic Reviews and Meta-analysis) (S1 PRISMA Checklist [15]). The protocol has been published and is available at: http://www.crd.york.ac.uk/PROSPERO/display_record.asp? [ID = CRD42014008919] (S1 Protocol).

### Acquisition of Data

On March 15^th^, 2014 we conducted a literature search using the metasearch engine “Mettā” (accessible at http://mengs1.cs.binghamton.edu/metta/search.action ) [16]. Mettā is a query interface that helps systematic reviewers to retrieve, filter, and assess articles from five leading medical databases: PubMed, EMBASE, CINAHL, PsycINFO, and the Cochrane Central Register of Controlled Trials. Medical Subject Headings (MeSH) terms used for key and text word searches included “Hepatitis C” OR “Hepatitis C Antibodies” OR “Hepatitis C Virus” AND “Point-of-Care Tests” OR “Rapid Test” OR “Rapid Assay”. We also searched the bibliographies and reference lists of eligible papers and related reviews, consulted experts in the field, and contacted several authors from the included articles to locate additional studies. The titles and abstracts of all of the articles identified in the primary search were evaluated, and we established a list of eligible potential studies for consideration in the full-text review. Studies that fulfilled all of the selection criteria were included in the systematic review and meta-analysis.

### Criteria for Inclusion of Studies in the Meta-Analysis

To recreate the 2×2 diagnostic table for estimating test accuracy, we included studies that employed point-of-care tests to detect anti-HCV (i.e., the index test), compared the results with a reference standard, and reported the results. A point-of-care test was defined as any commercially available assay that identified anti-HCV at or near the site of patient care. The test had to have a quick turnaround time (less than 30 min), allow for easy sampling, execution and reading of results, and have no requirement, or a minimal requirement, for cold chain and specialized equipment. The test results had to be available to the patient, physician and care team within an hour, which allowed for clinical management decisions to be made during the clinical encounter. Acceptable reference standards included a third-generation enzyme immunoassay (EIA), a microenzyme immunoassay, or a chemiluminescent immunoassay for the detection of anti-HCV. Two additional tests (recombinant immunoblast assay [RIBA] and nucleic acid testing) were considered to improve the reference standard [6]. We included studies of adults (>18 years old) published as abstracts or as full-text articles using any study design and conducted in any study settings (i.e., laboratory or field-based). Studies were not excluded based on sample size, study location, language of publication, or country of origin of the test. However, we excluded studies that dealt with the accuracy of laboratory-based tests, those with data that were unsuitable for recreating the 2×2 diagnostic table, reports from manufacturers and package inserts that could be subject to overt conflict-of-interest, and duplicate reports.

### Data Extraction

Two independent reviewers conducted the literature search, performed a quality assessment of the studies included in our analysis, and extracted the data necessary for estimating test accuracy. Any discrepancies were referred to a third reviewer, who adjudicated any unresolved discrepancies. The following information was extracted from each study: author, year of publication, location of study, index test (one or more), reference standard, study design, source of sera, sample size, the characteristics of the population sampled for sera collection, the cross-reactive sera included in panel, and the analytical sera included in evaluation of test performance. Detailed information about the index test was extracted from each study, including: the name of the test, the country of origin and the name of the manufacturer, the time required to read the results, the specimen type (serum, plasma or blood) sampled for the test, the volume of the sample (μL) needed for the test, the storage conditions for maintaining the test kit, special equipment needed (if any) to perform the test, and the shelf life of the test kit.

### Quality Assessment

We assessed the quality of the studies using the checklist of the QUADAS-2 (Quality Assessment of Diagnostic Accuracy Studies) tool [17] and STARD (Standards for the Reporting of Diagnostic Accuracy Studies) [18]. The QUADAS-2 sheet was completed following stepwise guidelines to assess the risk of bias (in four domains) and any concerns about applicability (in three domains) in each study. The STARD checklist consisted of 25 questions that were weighted equally (yes = 1, no = 0). The total score (out of 25) was calculated for each study.

### Data Synthesis

Data were extracted to construct 2×2 tables (reference test results vs. index test results (S1 Table). We defined anti-HCV positive as those subjects with disease and anti-HCV negative as those without disease, based on the reference test results. The index test results were reported as a true positive, a false positive, a false negative, or a true negative. Valid inconclusive index test results were combined with either false negative results (i.e., sera that were anti-HCV positive according to the reference test and inconclusive by index test) or false positive results (i.e., sera that were anti-HCV negative according to the reference test and inconclusive by index test) [13].

### Statistical Analysis

To estimate test accuracy, we calculated the sensitivities and specificities, positive and negative likelihood ratios (LRs), and the diagnostic odds ratios (DOR), along with 95% confidence intervals (CIs) (S1 File) [19]. We pooled test estimates using a bivariate random-effects regression model [20]. The model was used to draw hierarchical summary receiver operating characteristic (HSROC) curves. The curve of a test is a graph of sensitivity plotted against specificity that is obtained by varying the positivity threshold across all possible values. The graph plots sensitivity (the true positive rate) against 1- specificity (the false positive rate). To assess the levels of heterogeneity among test estimates, we calculated the inconsistency index (I^2^) [14]. We considered values of ≤ 25%, > 25–50%, > 50–75%, and > 75% to represent low, moderate, substantial, and considerable statistical heterogeneity, respectively. Multiple variables were selected a priori, and examined to investigate the potential sources of heterogeneity. Summary estimates and 95% CIs for each covariate were generated and compared in the meta-regression model. The DOR summarizes test accuracy as a single number, and can be used in a subgroup/meta-regression model to derive statistical values. A P value below 0.05 for the DORs was considered to be a significant difference among the levels of a particular covariate [21]. In order to compare the relative efficacy of tests, we pooled the estimates of individual tests that had three or more data points, and compared the estimates of individual tests with each other in the meta-regression model [21].

We performed all statistical analysis using the software program Meta-Analyst (Tufts Medical Centre, Boston, MA) [22].

## Results

### Literature Search & Study Characteristics

**Fig 1:**
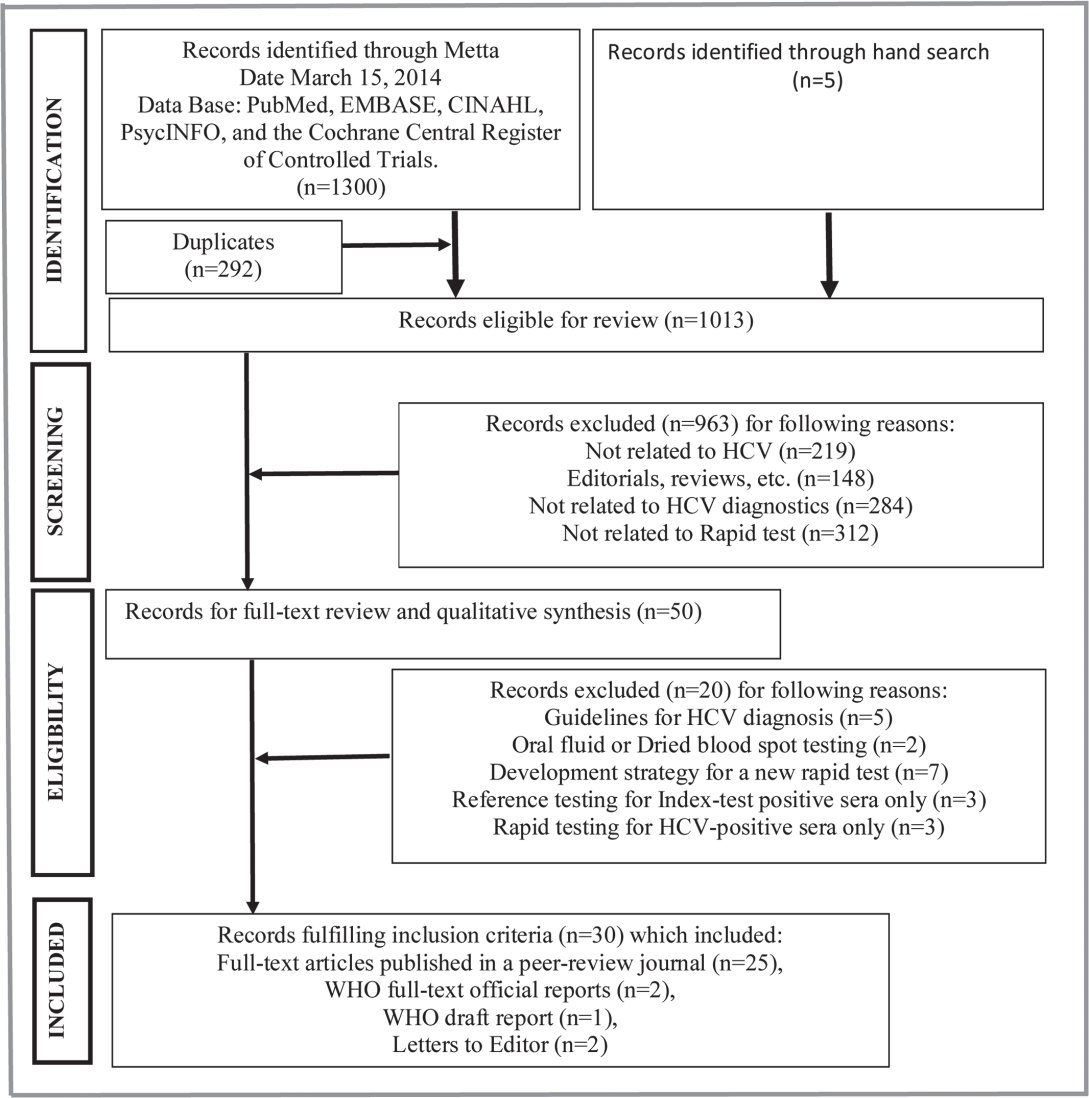
Flow diagram of study selection. List of 20 full-text excluded articles, with the reasons for exclusion shown in S2 Table.

Our search returned 1300 reports, of which 30 satisfied all of the inclusion criteria (Fig. 1) [12, 23–51]. Twenty full-text articles were excluded for the reasons shown in S2 Table. Twenty-five studies were published full-length manuscripts in peer-reviewed journals [12,23–28,33,34,36–51], two studies were published official reports [29,30], one was a draft report of the WHO [31], and two studies were published letters to the editor [32,35]. One study was published in Spanish [27] and the remaining 29 studies were in English. Sixteen studies were conducted in developing countries [23,24,26,28,32–37,43,44,47–49,51] and 14 studies were conducted in developed countries. The samples of 11 studies were collected from healthy blood donors, the general population, or from low-risk populations [12,24,29–31,35,37,38,48,50,51]. Those from a further 17 studies were taken from hospital inpatients, medical and surgical clinic patients, or high-risk populations. The nature of the patient population was not known in two studies [34,44]. The sample size in each study varied from 60 to 2754 subjects. In 17 studies, the type of specimen tested was serum alone [12,23,24,26–31,33,36,40,44,46–49]. Plasma alone was tested in three studies [34,50,51], whole blood alone was tested in 5 studies [25,32,35,37,45], oral fluid alone was tested in one study [42], oral fluid plus serum was tested in one study [43], and oral fluid plus whole blood was tested in one study [41]. The remaining two studies tested other combinations of those specimens [38,39] (Table 1).

**Table 1:** Details of the included studies.

**Study**	**Location**	**Reference standard**	**Index test**	**Specimen**	**Sample size**	**Study design**	**Patient population**
**Poovorawari et al 1994 [23]**	Thailand	ELISA 2^nd^, RIBA	Spot	Serum	192	Case-control	Hospital samples
**Mvere et al 1996 [24]**	Zimbabwe	EIA 2^nd^, INNO-LIA HCV Ab III	Spot	Serum	206	Cross-sectional	Blood bank
**Montebugnoli et al 1999 [25]**	Italy	ELISA (Ortho HCV 3.0), RIBA	Rapid	Whole blood	100	Case-control	Hospital samples
**Kaur et al 2000 [26]**	India	EIA 3rd	BiDot	Serum	2754	Cross-sectional	Hospital samples
**Buti et al 2000 [27]**	Spain	Immunometric technique, RIBA	Multiplo	Serum	188	Case-control	Hospital samples
**Yuen et al 2001 [28]**	China	EIA, PCR	SM-HCV	Serum	290	Case-control	Hospital samples
**WHO 2001 [29]**	Switzerland	ELISA, RIBA, PCR	α	Serum	257	Case-control	WHO panel
**WHO 2001 [30]**	Switzerland	ELISA, RIBA, PCR	TriDot 4^th^, Genedia	Serum	257	Case-control	WHO panel
**WHO 2002 [31]**	Switzerland	ELISA, RIBA, PCR	SDBioline	Serum	257	Case-control	Asia, Africa, Latin America & Europe
**Hui et al 2002 [32]**	Hong Kong	EIA	SM-HCV	Whole blood	197	Case-control	Hepatology clinic
**Daniel et al 2005 [33]**	India	EIA, RIBA, PCR	TriDot	Serum	2590	Cross-sectional	Outpatient /hepatology
**Scheiblauer et al 2006 [12]**	USA	EIA, PCR	β	Serum	381	Case-control	ICBS panel
**Njouom et al 2006 [34]**	Cameroon	EIA 3^rd^, PCR	ImmunocombHexagon	Plasma	329	Case-control	NK
**Torane et al 2006 [35]**	India	ELISA	Spot	Whole blood	60	Case-control	Blood banks
**Nyirenda et al 2008 [36]**	Malawi	ELISA, CLIA, Lineimmunoassay	Spot	Serum	202	Cross-sectional	Outpatient clinics
**Ivantes et al 2010 [37]**	Brazil	CLIA	Bioeasy	Whole blood	71	Cross-sectional	General population
**Lee et al 2010 [38]**	USA	EIA, RIBA	OraQuick	£	572	Cross-sectional	Low-risk persons
**Lee et al 2011 [39]**	USA	EIA, RIBA, PCR	OraQuick	£	2176	Case-control	Medical clinics
**Smith et al 2011 [40]**	USA	CLIA, RIBA	¥	Serum	1081	Cross-sectional	Injection drug users
**Smith et al 2011 [41]**	USA	MEIA/EIA/CLIA, RIBA	¥	Whole blood, Oral fluid	μ	Cross-sectional	Injection drug users
**Drobnik et al 2011 [42]**	USA	EIA, RIBA	OraQuick	Oral fluid	484	Cross-sectional	High-risk groups
**Cha et al 2012 [43]**	Korea	EIA	OraQuick	Oral fluid Serum	437	Case-control	Hospital samples
**Maity et al 2012 [44]**	India	ELISA	TriDot	Serum	300	Case-control	NK
**Jewett et al 2012 [45]**	USA	ELISA, RIBA, PCR	Chembio, Multiplo	Blood	406	Case-control	Injection drug users
**Kant et al 2012 [46]**	Germany	Architect HCV	Onsite	Serum	185	Case-control	Hospital samples
**Kim et al 2013 [47]**	Korea	ADVIA Centaur RIBA	SDBioline Genedia	Serum	100	Case control	Hospital samples
**Al-Tahish et al 2013 [48]**	Egypt	ELISA 3^rd^, HCV RNA	One-step TriDot 4^th^ Immunocomb	Serum	100	Case-control	Blood donors
**da Rosa et al 2013 [49]**	Brazil	Architect HCV test, PCR	Bioeasy	Serum	307	Case-control	Hepatology clinic
**O'Connell et al 2013 [50]**	USA	ELISA 3^rd^	€	Plasma	674	Case-control	Blood banks
**Tagny et al 2014 [51]**	Cameron	ELISA	Hexagon	Plasma	1998	Cross-sectional	Blood banks

NK = not known. α = Advanced, TriDot, Serodia, Spot, SeroCard. β = Acon, HepaScan, TriDot, Genedia, i+Lab, Dipstick, Assure, SPAN, ImmunoRAPIDO. ¥ = Chembio, Multiplo, OraQuick. € = OraQuick, Instant, Axiom, FirstVue. μ = 197, 282, 389, 285, 432, 265, 266. £ = Oral fluid, whole blood, finger stick, plasma, serum.

The index test characteristics are listed in Table 2. Thirty studies used 30 test brands that generated 73 data points. A single data point was generated by each of 16 tests. Two data points were generated for each of six tests (SPAN, ImmunoRAPIDO, SM-HCV, Bioeasy, Immunocomb and, Hexagon), and three or more data points were generated for each for seven tests (OraQuick, Genedia, SDBioline, TriDot, Chembio, Spot, and Multiplo). Estimates of TriDot and TriDot 4^th^ were similar and combined together. The number of data points generated for each specimen type were: serum (42 data points), whole blood/finger stick (13 data points), plasma (nine data points), and oral fluid (nine data points).

**Table 2:** Test specifications.

**Short test Name**	**Detailed test name**	**Manufacturer**	**Antigens**	**Time to test**	**Specimen to test**
**ACON**	ACON HCV test	ACON Laboratory, San Diego, CA, USA	-	-	Serum, plasma
**Advanced**	Advanced Quality One Step HCV Test	Bionike, Inc., San Francisco, USA	-	6 min	Whole blood, plasma, serum
**Assure**	Assure HCV Rapid test	MP Biomedical, LLC, CA, USA	-	-	Serum, plasma
**Axiom**	Axiom HCV Card	Axiom Diagnostics, Bierstadt, Germany	-	-	-
**BiDot**	HCV Bi-Dot	J. Mitra, New Delhi, India	Core, NS3, NS4, NS5	3 min	Serum, plasma
**Bioeasy**	HCV Rapid test Bioeasy	Bioeasy Diagnostica, Belo Honzonte, Minas Gerais, Brazil	Core, NS3, NS4, NS5	15–20 min	Whole blood, serum, plasma
**Chembio**	Chembio DPP HCV TEST	Chembio Diagnostics Systems, Inc, Mitford, New York, USA	Core, NS3, NS4, NS5	15–30 min	α
**CORE**	CORE HCV test	CORE Diagnostics, Birmingham, United Kingdom			
**Dipstick**	Entebbe Anti-HCV Dipstick	Labratorium Hepatika Mataram, Indonesia	-	-	-
**FirstVue**	First HCV	AT First Diagnostics, LLC, Woodbury, NY	-	-	-
**Genedia**	Genedia HCV Rapid LF	Green Cross Life Sciences Corp., Korea	Core, NS3, NS4, NS5	20–30 min	Whole blood, serum, plasma
**HepaScan**	Hepa-Scan HCV Card test	Bhat Biotech India Pvt Ltd. Bangalore, Karnataka, India	-	-	-
**Hexagon**	Hexagon HCV test	Human Gesellschaft fur Biochemica und Diagnostica μbH, Wiesbaden, Germany	Core, NS3, NS4, NS5	5–20 min	Serum, plasma, whole blood
**i+Lab**	i+Lab anti-HCV test	i+MED, Lab Company Ltd. Bangkok, Thailand	-	-	-
**Immunocomb**	ImmunoComb II HCV test	Inverness Medical Innovations, Waltham, MA, USA	-	10–15 min	Serum, plasma
**Immuno-RAPIDO**	Immuno-Rapido HCV	Wama Diagnostica, Sao Carlos, Brazil	-	-	-
**Instant**	Instant View Cassette	Alfa Scientific Designs Inc., Poway CA, USA	-	-	-
**Multiplo**	Multiplo Rapid HIV/HCV Antibody Test	MedMira Lab Inc., Halifax, Nova Scotia, Canada	Core, NS3	3 min	Whole blood, plasma, serum
**One-step**	One Step HCV Rapid Test	Inter-chemical Ltd., Shenzhen, China	-	10 min	Whole blood, serum, plasma
**Onsite**	Toyo anti-HCV test	Turklab, Izmir, Turkey	-	5–15 min	β
**OraQuick**	OraQuick Rapid Antibody test	OraSure technologies, Bethlehem, PA, USA	Core, NS3, NS4	20–40 min	β
**Rapid**	Anti-HCV Antibody Rapid test	Tema Recerca, Bologna, Italy	-	3 min	Whole blood
**SDBioline**	SDBioline HCV test	SD Standard Diagnostics Ltd., Koyanagi-do, Korea	Core, NS3, NS4, NS5	5–20 min	Serum, plasma, whole blood
**SeroCard**	SeroCard HCV	Trinity Biotech plc., Bray, Ireland	-	19 min	Serum, plasma, whole blood
**Serodia**	Serodia HCV	Fuji Rebio Inc., Tokyo, Japan	-	2 hr 45 min	Serum, plasma
**SM-HCV**	SM-HCV Rapid Test	SERO-Med Labor Spezialitaten Pollen Feld, Germany	Core, NS3, NS4	3 min	Serum, whole blood
**SPAN**	Signal HCV	Span Diagnostics Ltd. Surat, India	-	-	-
**Spot**	GLD HCV-SPOT	Genelabs Diag. Pty, Ltd., Singapore	-	10 min	Serum, plasma
**TriDot**	HCV TriDot Rapid	J. Mitra and Co. Ltd., New Delhi, India	Core, NS3, NS4, NS5	9 min	Serum, plasma
**TriDot 4th**	HCVComb TriDot 4^th^	J. Mitra and Co. Ltd., New Delhi, India	-	-	-

- = not specified in reference. α = Whole blood, serum, plasma, oral fluid. β = Whole blood, finger stick, serum, plasma, oral fluid.

### Study Quality

Ten studies employed a cross-sectional design [24,26,33,36–38,40–42,51], and 20 studies were case-controls. Nine studies used EIA alone as the reference standard [26,32,35,37,43,44,46,50,51]. Twenty-one studies used EIA in combination with RIBA or/ PCR. All of the studies administered the same reference test to all patients, thus avoiding partial or differential verification biases. Four studies reported that the index test readers were blinded [24,25,32,50]. The other studies made no reference to blinding of the index test readers. One study tested all samples twice [28]. The results of the index tests were independently read by more than one technician in five studies [12,29,30,40,41]. Eight studies received funding from, or reported another financial relationship with the pharmaceutical industry [25,28,34,38,42,46,48,49]. Nine studies received test kits from manufacturers [23,29,30,37,40,41,45,51], and five studies were funded by non-profit official organizations [33,36,43,44,50]. Seven studies explicitly declared no conflict of interest [41,43–45,49–51]. The remaining studies made no disclosure of conflict of interest.

The QUADAS-2 sheet reveals any major risk of bias in patient selection, index test, or reference standard (Table 3). Among the included studies, bias in patient selection resulted from either i) a case-control study design, or ii) a poor description of patient selection and clinical scenario. Bias in the index test was primarily due to a lack of reported blinding while reading test results. Bias in the reference standard was due to the inability to use a Center for Disease Control (CDC)-recommended reference standard (EIA plus RIBA and/or nucleic acid testing). The quality of study reporting ranged from poor to good (i.e., STARD scores from 8 to 23, out of a maximum possible score of 25), with a number of items missing from reporting of diagnostic accuracy.

**Table 3:** QUADAS-2 assessments and STARD scores.

	**QUADAS 2 SHEET**							**STARD**
**Study**	**Patient selection**		**Index test**		**Reference standard**		**Flow and timing**	**Score**
	**Risk of bias**	**Concern about applicability**	**Risk of bias**	**Concern about applicability**	**Risk of bias**	**Concern about applicability**	**Risk of bias**	**Total (out of 25)**
**Poovorawari 1994 [23]**	LR	LR	UC	LR	LR	LR	LR	12
**Mvere 1996 [24]**	HR	LR	LR	LR	LR	LR	LR	13
**Montebugnoli 1999 [25]**	HR	LR	LR	LR	LR	LR	LR	12
**Kaur 2000 [26]**	LR	LR	UC	LR	HR	LR	LR	11
**Buti 2000 [27]**	UC	LR	UC	LR	LR	LR	LR	15
**Yuen 2001 [28]**	HR	LR	LR	LR	LR	LR	LR	11
**WHO 2001 [29]**	HR	LR	LR	LR	LR	LR	LR	23
**WHO 2001 [30]**	HR	LR	LR	LR	LR	LR	LR	23
**WHO 2002 [31]**	Draft report, data insufficient for evaluation							
**Hui 2002 [32]**	HR	LR	LR	LR	HR	LR	LR	17
**Darniel 2005 [33]**	LR	LR	LR	LR	LR	LR	LR	15
**Scheiblauer 2006 [12]**	HR	LR	LR	LR	LR	LR	LR	23
**Njouom 2006 [34]**	HR	LR	UC	LR	LR	LR	LR	10
**Torane 2006 [35]**	HR	LR	UC	LR	HR	LR	LR	10
**Nyirenda 2008 [36]**	LR	LR	UC	LR	LR	LR	LR	15
**Ivantes 2010 [37]**	LR	LR	UC	LR	HR	LR	LR	15
**Lee 2010 [38]**	LR	LR	UC	LR	LR	LR	LR	8
**Lee 2011 [39]**	HR	LR	UC	LR	LR	LR	LR	20
**Smith 2011 [40]**	LR	LR	LR	LR	LR	LR	LR	19
**Smith 2011 [41]**	LR	LR	LR	LR	LR	LR	LR	17
**Drobnik 2011 [42]**	LR	LR	UC	LR	LR	LR	UC	13
**Cha 2012 [43]**	HR	LR	UC	LR	HR	LR	LR	14
**Maity 2012 [44]**	HR	LR	UC	LR	HR	LR	LR	14
**Jewett 2012 [45]**	HR	LR	UC	LR	LR	LR	LR	15
**Kant 2012 [46]**	HR	LR	UC	LR	HR	LR	LR	14
**Kim 2013 [47]**	HR	LR	UC	LR	LR	LR	LR	17
**Al-Tahish 2013 [48]**	HR	LR	UC	LR	LR	LR	LR	14
**da Rosa 2013 [49]**	HR	LR	UC	LR	LR	LR	LR	16
**O'Connell 2013 [50]**	HR	LR	LR	LR	HR	LR	LR	12
**Tagny 2014 [51]**	LR	LR	UC	LR	HR	LR	LR	16

LR = low-risk, HR = high-risk, UC = unclear

### Pooled Test Accuracy

**Fig 2:**
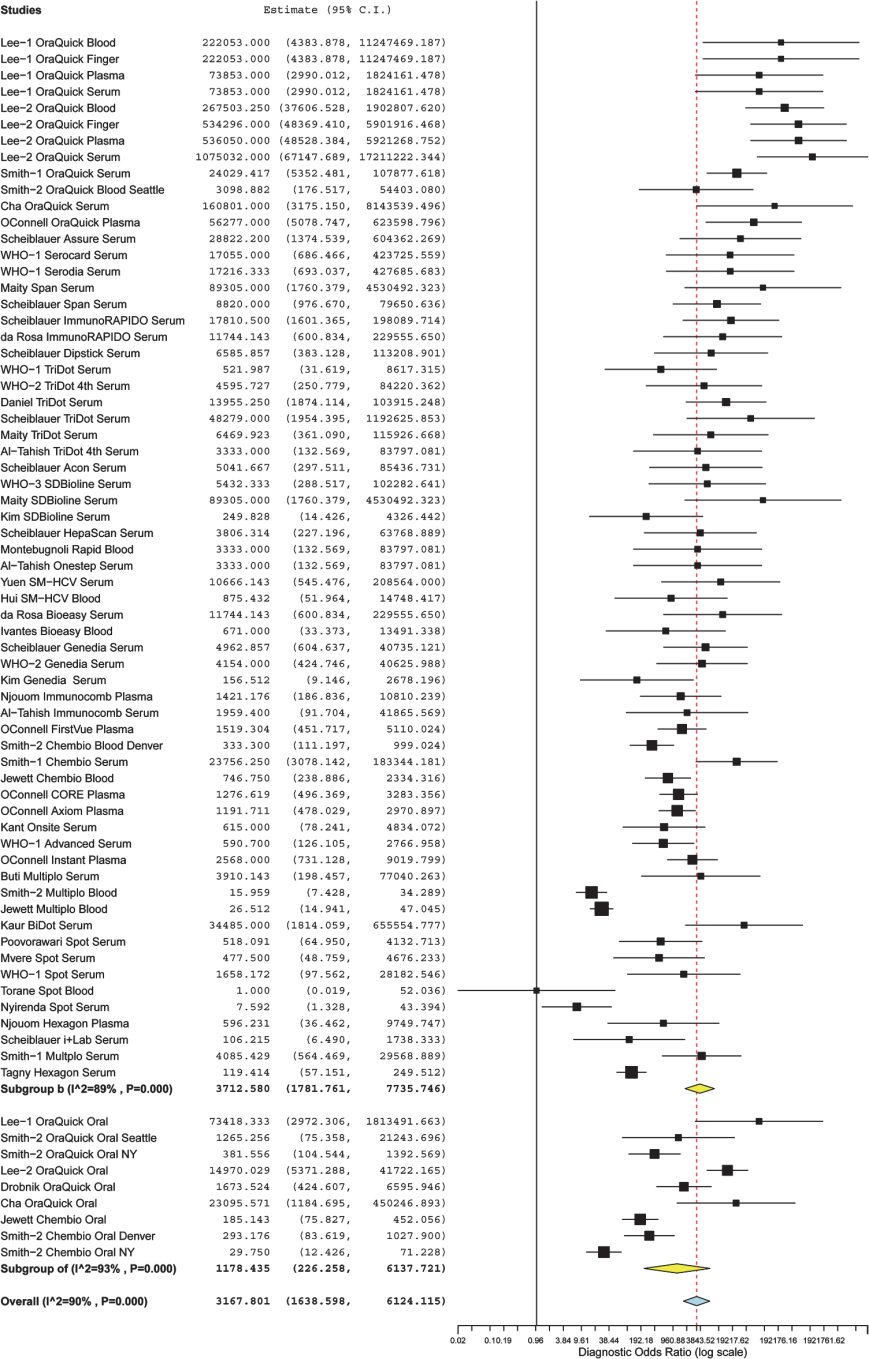
Forest plot of the diagnostic odds ratio on a log scale and 95% confidence intervals (CIs) of 30 studies stratified by specimen type. Whole blood, finger stick, plasma, and serum samples generated 64 data points (subgroup B) and oral fluid samples generated 9 data points (subgroup OF). Diagnostic odds ratios for each data point are shown as solid squares. Solid lines are 95% CIs. Squares are proportional to the weights based on the random effect model. Pooled estimates and 95% CIs are denoted by the diamond at the bottom of each subgroup list. I^2^ and P values indicate the heterogeneity of the studies.

**Fig 3:**
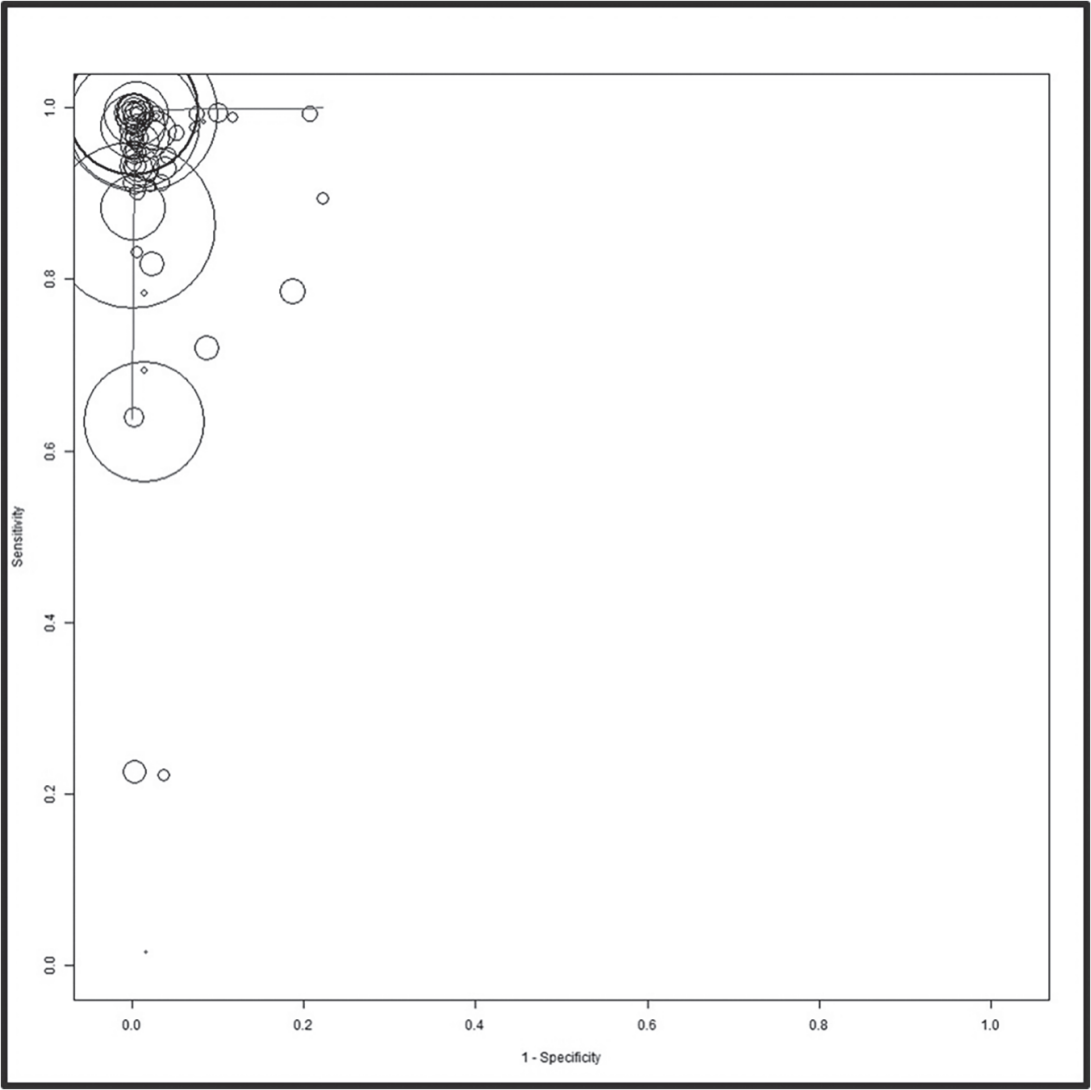
The summary receiver operating characteristic (SROC) plot based on a bivariate random effects model. The sensitivities and specificities of rapid point-of-care tests with the reference test for Anti-HCV are shown. The sensitivity of a test is plotted against 1-specificity, allowing the comparison of multiple tests at the same time. The circles represent single test data points from individual studies. The sizes of the circles are proportional to the number of patients included in the study. The curved line is the regression of the ROC curve summarizing the overall diagnostic accuracy.

A forest plot of the sensitivity and specificity estimates and 95% confidence intervals (CIs) for the 30 studies, stratified by their test brands, is shown in S1 Figure. Tests were ranked in descending order based on the test estimates. The sensitivity (range: 16.0–99.9%) and specificity (range: 77.8–99.7%) of individual tests were heterogeneous and varied widely. The overall pooled sensitivity, specificity, positive LR, negative LR, and DOR for all tests were 97.46% (95% CI: 95.92–98.43), 99.58% (99.28–99.75), 80.17 (55.35–116.14), 0.03 (0.02–0.04), and 3032.85 (1595.86–5763.78) respectively (Fig. 2, S2 Figure). This suggested a high pooled accuracy among all studies. The ROC curve also indicated high sensitivity with high specificity, as the curve approached the upper left hand corner of the graph where both measures equal 1 (Fig. 3).

### Sources of Heterogeneity

We found substantial heterogeneity among studies in the pooled sensitivity (I^2^ = 94%), specificity (I^2^ = 88%), positive LR (I^2^ = 89%), negative LR (I^2^ = 90%), and DOR (I^2^ = 90%) (Table 4). Multiple covariates were used in subgroup analysis and tested statistically in the meta-regression model to determine the reason(s) for the heterogeneity. None of the following factors investigated accounted for the heterogeneity: specimen tested (whole blood, plasma, serum or oral fluid), year of publication (before vs. after 2005), location of study (developed vs. developing countries), reference standard (EIA alone vs. EIA with a recombinant immunoblast assay or nucleic acid test), study design (cross-sectional vs. case-control), source of sera (blood banks vs. hospitals/clinics), or study quality (STARD score high, >15 vs. low, ≤15). Studies conducted in developed and developing countries both had high pooled accuracy values; however, DORs were 4.8 times higher in studies conducted in developed countries (5348.36, 95% CI: 2332.97–12261.18) compared to developing countries (1113.39, 95% CI: 450.40–2752.33; P = 0.015). None of the other variables had a significant effect on test performance.

**Table 4:** Accuracy estimates from subgroup analysis.

**Variable**	**Subgroups (data points)**	**Pooled Sensitivity (95% CI), %**	**Pooled Specificity (95% CI), %**	**Positive LR (95% CI)**	**Negative LR (95% CI)**	**DOR (95%CI)**	**I** ^2^ **¥**	**P value €**
**Year**	Prior to 2005 (15)	96.5 (93.4–98.2)	98.0 (95.7–99.0)	46.01 (22.69–93.31)	0.019 (0.012–0.031)	2191.76 (1055.31–4552.04)	15%	Reference
	After 2005 (58)	95.8 (93.9–97.1)	99.0 (98.5–99.3)	91.02 (59.72–138.72)	0.031 (0.019–0.049)	3117.86 (1494.36–6505.14)	91%	0.87
**Location of test**	Developing countries (27)	91.6 (87.0–94.7)	98.4 (97.4–99.1)	52.96 (31.41–89.31)	0.055 (0.027–0.115)	1113.39 (450.40–2752.33)	85%	Reference
	Developed countries (46)	97.4 (96.0–98.4)	99.1 (98.4–99.5)	100.67 (59.74–169.62)	0.020 (0.013–0.030)	5348.36 (2332.97–12261.18)	90%	0.02
**Fluid**	Serum (42)	96.5 (93.9–98.0)	99.0 (98.4–99.4)	93.81 (56.93–154.56)	0.026 (0.016–0.043)	3927.64 (1800.18–8569.35)	80%	Reference
	Whole blood (13)	94.7 (89.1–97.5)	98.6 (96.3–99.4)	55.56 (20.80–148.43)	0.03 (0.01–0.09)	1674.60 (268.43–10446.91)	95%	0.21
	Plasma (9)	97.6 (93.0–99.2)	99.0 (97.3–99.6)	93.78 (35.68–246.49)	0.017 (0.007–0.039)	4447.22 (1451.08–13629.73)	78%	0.83
	Oral fluid (9)	93.9 (89.7–96.4)	98.6 (96.3–99.6)	66.02 (18.88–230.81)	0.070 (0.037–0.131)	1178.43 (226.25–6137.72)	93%	0.16
**Ref test**	Confirmatory tests-RIBA/PCR (57)	96.2 (94.4–97.5)	98.9 (98.3–99.3)	85.71 (53.92–136.23)	0.027 (0.017–0.043)	3377.26 (1478.29–7716.04)	91%	Reference
	EAI/ELISA alone (16)	95.2 (91.8–97.2)	98.6 (97.6–99.3)	62.00 (34.97–109.94)	0.035 (0.018–0.071)	2158.42 (852.59–5464.29)	81%	0.82
**Study design**	Case-control (46)	95.7 (93.1–97.3)	98.5 (97.7–99.0)	56.67 (39.22–87.77)	0.030 (0.019–0.048)	2258.37 (1141.47–4468.10)	82%	Reference
	Cross-sectional (27)	96.4 (94.2–97.8)	99.2 (98.5–99.6)	116.99 (58.58–233.65)	0.078 (0.043–0.140)	4968.90 (1385.21–14824.01)	94%	0.36
**Source of sera β**	Hospital/clinics (34)	95.1 (92.7–96.7)	98.9 (98.0–99.4)	83.50 (45.60–152.87)	0.037 (0.0020–0.068)	2499.08 (868.79–7188.56)	94%	Reference
	Blood banks (34)	96.8 (93.6–98.4)	98.9 (98.2–99.4)	83.10 (48.30–142.99)	0.023 (0.013–0.040)	3518.01 (1697.79–7289.75)	74%	0.46
**Score α**	<15 (29)	97.0 (94.8–98.3)	99.0 (98.3–99.)	89.91 (52.81–153.06)	0.022 (0.014–0.035)	3990.06 (1936.62–8220.83)	70%	Reference
	≥15 (43)	95.3 (92.7–97.0)	98.8 (97.9–99.3)	70.98 (43.09–116.93)	0.035 (0.002–0.06)	2275.58 (930.61–5564.35)	92%	0.243

LR = Likelihood ratio, DOR = Diagnostic Odds Ratio, I^2^¥ = inconsistency index test for heterogeneity (<25 = low, >25 to 50% = moderate; >50 to 75% = substantial, & >75% = considerable statistical heterogeneity), € = P value determined from meta-regression model, α = score of one study not calculated, β = source of sera from five studies not known.

### Qualitative Analysis

Four studies evaluated the impact of HCV genotype on index test performance [12,29,30,33] (S3 Table). These studies found that HCV genotype diversity had no or minimal influence on the performances of index tests. Three studies evaluated the performances of four index tests (Spot, OraQuick, Chembio, and Multiplo) in HCV sera panels, admixed with HIV cross-reactive sera [36,40,41] (S4 Table). OraQuick was the only index test brand that did not show false positive results in HCV-reactive sera, or oral fluid samples that were cross-reactive for HIV. In addition, OraQuick did not interfere with the HCV positivity of oral fluid by interacting with the conditions in the mouth, chemical substances (bilirubin, hemoglobin, lipids, etc.), or conditions of storage or testing (i.e., hot storage, cold storage, or hot testing). Eight studies evaluated 21 tests (TriDot, TriDot 4^th^, Advanced, Serodia, Spot, SeroCard, Genedia, Acon, Hexagon, HepaScan, i+Lab, Dipstick, Assure, SPAN, ImmunoRAPIDO, OraQuick, SDBioline, CORE, Instant View, Axiom, FirstVue) against seven commercially available seroconversion panels [12,29,30,38,39,43,47,50] (S5 Table). OraQuick, evaluated by four studies, was the only test that detected HCV antibodies in seroconversion panels earlier than the reference test; it had the most consistent performance compared to the reference test [38,39,43,50]. All of the other tests detected HCV antibodies in seroconversion panels later than the reference test. Eight tests (Serodia, SeroCard, Genedia, TriDot 4^th^, CORE, Instant view, AXIOM and FirstVue) failed to pick up one or more positive panels, and/or showed inconsistent panel results.

### Individual Test Accuracy

Seven tests (OraQuick, Genedia, SDBioline, TriDot, Chembio, Spot, and Multiplo) generated three or more (range: 3–12) data points each for tests of serum, plasma, and whole blood. OraQuick and Chembio generated six and three data points each, respectively, for tests of oral fluids (Table 5). The estimates for these tests were evaluated statistically in the meta-regression model. OraQuick’s performance in tests of serum, plasma and whole blood (12 data points) showed the highest pooled sensitivity and specificity and moderate heterogeneity among studies (I^2^ = 36%). The pooled DOR of OraQuick was significantly higher than that of the other six tests. After OraQuick, the pooled DOR for TriDot and SDBioline were the next highest, followed by those of Genedia and Chembio. Spot and Multiplo tests showed poor sensitivity estimates (S3 Figure).

**Table 5:** Accuracy estimates of 7 tests with three or more data points evaluated by the meta-regression model.

**No**.	**Test**	**Sample**	**Data point**	**Pooled sensitivity% (95% CI)**	**Pooled specificity % (95% CI),**	**Positive LR (95% CI)**	**Negative LR (95% CI)**	**DOR (95%CI)**	**I** ^2^ **¥**	**P value €**
**1**	**OraQuick**	Whole blood, plasma, serum	12	99.5 (98.9–99.8)	99.8 (99.6–99.9)	445.84 (268.97–739.00)	0.004 (0.002–0.006)	116693.58 (44452.79–306333.78)	36%	Reference
**2**	**TriDot α**	Serum	6	98.2 (95.7–999.3)	98.4 (90.8–99.7)	61.22 (10.88–344.50)	0.009 (0.007–0.011)	6091.17 (1907.17–19454.14)	7%	<0.001
**3**	**SDBioline**	Serum	3	93.5 (73.2–98.7)	99.5 (97.7–99.9)	186.45 (37.80–919.53)	0.038 (0.004–0.402)	3995.08 (164.72–96896.06)	66%	0.003
**4**	**Genedia**	Serum	3	93.4 (63.5–99.1)	98.8 (96.9–99.5)	75.89 (30.30–190.08)	0.037 (0.008–0.170)	1781.13 (241.82–13118.77)	52%	<0.001
**5**	**Chembio**	Whole blood, serum	3	95.1 (89.8–97.8)	98.6 (95.0–99.6)	68.45 (18.16–257.97)	0.065 (0.035–0.120)	1491.76 (201.71–111032.24)	85%	<0.001
**6**	**Spot**	Whole blood, serum	5	75.4 (14.1–98.3)	95.2 (91.9–97.1)	14.38 (7.96–25.99)	0.072 (0.016–0.318)	92.51 (7.54–1135.06)	81%	<0.001
**7**	**Multiplo**	Whole blood, serum	4	85.9 (75.7–92.2)	96.1 (85.8–99.0)	20.22 (5.60–73.01)	0.260 (0.167–0.406)	178.95 (25.63–1249.02)	92%	<0.001
**8**	**OraQuick β**	Oral fluid	6	95.9 (92.1–97.9)	99.4 (98.1–99.8)	147.98 (47.92–456.92)	0.037 (0.020–0.067)	4254.017 (825.64–21918.185)	80%	<0.001 (1 vs. 8)
**9**	**Chembio β**	Oral fluid	3	88.0 (81.6–92.4)	94.0 (74.2–98.8)	14.93 (3.18–69.95)	0.141 (0.094–0.213)	112.61 (27.61–459.16)	83%	<0.001 (8 vs. 9)
										<0.001 (1 vs. 9)

α = TriDot and TriDot 4^th^ estimates clubbed together, β = estimates of OraQuick and Chembio obtained with oral fluid samples are evaluated separately, I^2^¥ = inconsistency index test for heterogeneity (<25 = low, >25 to 50% = moderate; >50 to 75% = substantial and >75% = considerable statistical heterogeneity), € = P value determined from meta-regression model.

The pooled estimates of OraQuick from tests of serum, plasma, and whole blood were significantly higher than those obtained from tests of oral fluid samples, while OraQuick had significantly higher test estimates for oral fluids than Chembio (S4 Figure).

Of the 23 tests that could not be evaluated in the meta-regression model, we identified nine that were unlikely to be useful in routine assays, based on the following parameters: sensitivity < 90% (BiDot, Hexagon, i+Lab), specificity < 95% (Onsite), −LR > 0.1(BiDot, FirstVue, Hexagon and i+Lab), +LR < 10 (Onsite), and poor performance in seroconversion panels (CORE, Instant, Axiom, and FirstVue) [19]. The DORs of the remaining 14 tests (range: 590.70–28822.20) are shown in order of their estimates to illustrate comparative test performance (S5 Figure).

## Discussion

The meta-analysis that we conducted has six major strengths, as it was based on: (i) a global and complete literature search, with strict inclusion criteria; (ii) the definition of a strategy to include and analyze inconclusive results; (iii) the use of a bivariate statistical model; (iv) a statistical comparison of summary estimates for diagnostic accuracy within subgroups using a meta-regression model, to make relevant conclusions; (v) the evaluation of heterogeneity and its potential sources in the meta-regression model; (vi) an assessment of the tests’ analytical sensitivity, which included the influences of genotype, cross-reactive sera, interfering substances, and seroconversion panels on the performances of various test brands. We found sufficient information to enable an evaluation of the performances of several individual tests, and hence disagree with an earlier report that stated that the accuracy of individual tests cannot be evaluated due to lack of data [11]. We believe a bivariate model was appropriate for our meta-analysis for three reasons. First, our reference standards were enzyme immunoassays that employed consistent standard positive thresholds across the studies, as per the manufacturer’s guidelines. Second, index tests yielded positive and negative results as consistent cut-offs on the device (appearance of a colored line or dot), across the studies. Finally, all studies administered the same reference standard to all patients, thereby avoiding partial or differential biases [52].

The reference standard used by each study was not found to influence the accuracy of the index test. Third generation enzyme immunoassays incorporate HCV antigens from the core, NS3, NS4, and NS5, and have a diagnostic sensitivity and specificity of >99% [53]. The CDC has recommended RIBA as an additional, more specific test for the detection of anti-HCV in serum or plasma specimens that have been found reactive in ELISA [6]. However, RIBA assays have low sensitivity, they require high costs, complex manual attention, and a long time for processing, and they do not differentiate active infection from past exposure with spontaneous clearance [53]. Manufacturers have therefore discontinued RIBA HCV, and the CDC has updated the algorithm for HCV diagnosis to recommend nucleic acid testing for HCV RNA in subjects who test anti-HCV positive [7].

Our meta-analysis showed high pooled accuracy in point-of-care tests for the detection of anti-HCV. However, the sensitivities (range: 16.0–99.9%) and specificities (range: 77.8–99.7%) of individual tests were heterogeneous and varied widely. We must be cautious when evaluating the accuracy of tests, as our meta-analysis was subject to the detection, spectrum, and sampling bias of the original studies [14]. A case-control design in studies is apt to overestimate accuracy, with the potential for spectrum bias. In addition, only four studies explicitly mentioned blind reading of the index test results, suggesting that there could have been detection bias in the remaining studies. This could artificially inflate the sensitivity and specificity estimates of the index test. Lastly, there was substantial heterogeneity among studies in the pooled estimates, and none of the covariates tested were found to account for the observed heterogeneity.

In our meta-analysis, all of the tests performed better in studies that were conducted in developed countries than in developing countries. It is well-known that the performance characteristics of any test vary markedly with the prevalence of the condition in the population being assessed [54,55]. This should be kept in mind given that these tests are primarily meant to be employed in regions of the world where resources are limited. In addition, HCV has substantial genetic diversity, with six known genotypes, and HCV genotype is known to have a significant effect on diagnostic accuracy. Four studies in our analysis evaluated the impact of HCV genotype on index test performance [12,29,30,33], but this factor did not influence the performances of the assays.

During the evaluation of any of the tests that we considered, the impact of co-infections (with HIV, HBV, syphilis, etc.) on test performance must be included. Among them, HIV/HCV co-infection has special importance due to its significant burden worldwide, especially among injection drug users and within the Asia-Pacific region [56]. All three of the studies that addressed this issue found that false positive HCV results for Spot, Chembio, and Multiplo test brands were associated with HIV positivity. Data on this important issue are limited and further studies are needed to evaluate the influence of HIV, HBV, and syphilis co-infection on test performance. This issue will become even more important once multiplex point-of-care tests for HBV, HCV, and HIV are marketed for integrated HIV-sexually transmitted diseases screening programs [57].

Another consideration for the evaluation of test performance is the time at which the anti-HCV test becomes positive during the course of acute HCV infection. Eight studies used seroconversion panels, and determined the time difference (in days) between the first sample from the panel that was detected to be positive with the index test and that of the reference test. With the exception of OraQuick, all tests lagged behind the reference test in the detection of the first positive sample by a few days to a few weeks. Several tests performed poorly and failed to pick up one or more panels, and/or showed inconsistent results in some panels. Thus, when there is a high clinical suspicion of infection but a point-of-care test is negative, further testing should be conducted with a conventional laboratory test or nucleic acid test.

Based on the accuracy and analytical performance of OraQuick, we conclude that this test has the potential to be used as a rapid diagnostic test for HCV infection. The results obtained with it are comparable (and even possibly better) than those of third-generation enzyme immunoassays. In fact, the U.S. Food and Drug Administration (FDA) has approved OraQuick as the first rapid blood test for HCV antibodies in individuals 15 years and older [58]. Subsequent to this, in their update the CDC included OraQuick as the first-line rapid screening test for HCV infection using finger stick capillary blood and venipuncture whole blood [7]. The Clinical Laboratory Improvement Amendments [CLIA]-waiver provides wider testing access to persons at risk for HCV infection, permitting the use of the assay in physician offices, clinics, emergency rooms, and other counseling and testing sites.

It is essential for policymakers and hospital administrators to know that the performances of point-of-care tests for detection of anti-HCV vary widely. Amongst the seven tests evaluated in our meta-regression, OraQuick had the highest test sensitivity and specificity and showed better performance than a third generation enzyme immunoassay in seroconversion panels. The next highest test sensitivities and specificities were from TriDot and SDBioline, followed by Genedia and Chembio. The Spot and Multiplo tests had produced poor test sensitivities but high specificities. Nine of the remaining 23 tests produced poor test sensitivities and specificities and/or poor performances in seroconversion panels, while 14 tests had high test estimates with wide diagnostic odds ratios ranging from 590.70 to 28822.20. Many of these tests also had problems in the qualitative analysis, namely interference by cross-reactive HIV sera and/or poor performances in seroconversion panels.

Oral fluid-based point-of-care tests have significant advantages. Sampling does not require staff training in venipuncture, and noninvasive sampling would improve compliance and make HCV screening more acceptable [59]. However, such oral tests have lower sensitivities compared to blood-based tests. This is possibly due to a lower concentration of antibodies in oral fluid than in blood, or to dilution of the sample by the collection buffer. Also, oral fluid HCV positivity may be affected by oral pathology, or the collection of oral fluid after the use of mouth wash or acidic beverages by subjects. Lee et al [39] examined this possibility with the OraQuick test and found no interference with positive or negative oral fluid samples in the presence of fluids related to gingivitis, including bleeding gums, wearing of dentures, use of tobacco, etc. Similar studies are needed with other oral fluid-based point-of-care tests which have potential to be used as first-line tests in expanded screening initiatives. Blood-based tests could then be used to detect infections missed by oral fluid tests.

While the studies included in the present meta-analysis addressed accuracy of point-of-care tests for HCV antibodies, we found that little information was available pertaining to the evaluation of these tests for HCV-related, patient-centered outcomes. The most important information would be the feasibilities and outcomes of these tests in settings where anti-HCV testing is routinely conducted. Future studies are needed to assess how these tests perform in various situations, including: (i) seroepidemiologic studies in low and high endemic zones; (ii) screening of blood and blood products for anti-HCV and the prevention of transfusion-transmitted HCV infection; (iii) diagnosis and follow-up of acute hepatitis and fulminant hepatic failure; (iv) diagnosis of HCV-related chronic hepatitis and cirrhosis and the evaluation of anti-viral therapy. This information will be fundamental to shaping global diagnostic policies as they become more patient-centered over time. There is also a need for studies that address how accuracy varies with the prevalence of infection in different populations and settings.

Global hepatitis C treatment is evolving quickly, and there is an urgent need to increase screening for this infection in high-risk populations [60]. Point-of-care tests offer major benefits for screening and control of HCV infection, especially in the widely dispersed regions of the world that struggle with endemic poverty [10]. At present, practitioners are guided in the selection of individual tests only by manufacturers’ claims, which are expected to be biased. For the first time, our meta-analysis critically evaluated the performances of commercially available, individual point-of-care tests for hepatitis C. We found that the performances of individual tests varied widely. Health care policy makers, health administrators and laboratorian should consider these data in the selection of appropriate point-of-care tests for extended screening of hepatitis C in areas with limited financial resources.

## Supporting Information

S1 PRISMA Checklist (DOC)

S1 Figure Forest plot of the sensitivity and specificity estimates and 95% confidence intervals (CIs) of 30 studies stratified by test brands. Tests are placed in descending order based on the test estimates. Thirty studies had used 30 test brands and generated 73 data points. Estimates of TriDot and TriDot 4^th^ were similar and have been clubbed together. Estimates of two tests (OraQuick and Chembio) obtained on oral fluid testing are shown separately. Estimates of sensitivity and specificity from each study are shown as solid squares. Solid lines represent the 95% CIs. Squares are proportional to the weights based on the random effect model. Pooled estimates and 95% CIs is denoted by the diamond at the bottom. I^^2^ and p values represents heterogeneity of studies.
(TIF)

S2 Figure Forest plot of the sensitivity and specificity estimates and 95% confidence intervals (CIs) of 30 studies stratified by specimen collected for testing. Whole blood, finger stick, plasma, and serum samples had generated 64 data points (b) and oral fluid samples had generated 9 data points (of). Estimates of sensitivity and specificity from each study are shown as solid squares. Solid lines represent the 95% CIs. Squares are proportional to the weights based on the random effect model. Pooled estimates and 95% CIs is denoted by the diamond at the bottom. I^^2^ and p values represents heterogeneity of studies.
(TIF)

S3 Figure Forest plot of the diagnostic odds ratio on a log scale of 7 tests with ≥3 data points arranged in descending order. Diagnostic odds ratio from each data point are shown as solid squares. Solid lines represent the 95% CIs. Squares are proportional to the weights based on the random effect model. Pooled estimates and 95% CIs is denoted by the diamond at the bottom. I^^2^ and p values represents heterogeneity of studies.
(TIF)

S4 Figure Forest plot of the diagnostic odds ratio on a log scale of 2 tests with oral fluid as the test sample. Diagnostic odds ratio from each data point are shown as solid squares. Solid lines represent the 95% CIs. Squares are proportional to the weights based on the random effect model. Pooled estimates and 95% CIs is denoted by the diamond at the bottom. I^^2^ and p values represents heterogeneity of studies.
(TIF)

S5 Figure Forest plot of the diagnostic odds ratio on a log scale of 14 tests with less than three data points. Diagnostic odds ratio each data point are shown as solid squares. Solid lines represent the 95% CIs. Squares are proportional to the weights based on the random effect model. Pooled estimates and 95% CIs is denoted by the diamond at the bottom. I^^2^ and p values represents heterogeneity of studies.
(TIF)

S1 File Methodology document. Systematic review methodology & definitions of relevant accuracy estimates.
(DOCX)

S1 Protocol Protocol HCV meta-analysis. (PDF)

S1 Table 2×2 data table of included 30 studies evaluating 30 test brands and 73 data points. (DOCX)

S2 Table List of 20 full-text excluded articles, with the reasons for exclusion. (DOCX)

S3 Table Performance of Index tests in relation to HCV genotype diversity. (DOCX)

S4 Table Performance of Index tests for anti-HCV in relation to cross-reactive HIV sera, oral pathologies and conditions, biological substances and storage and testing conditions. (DOCX)

S5 Table Performance of Index test in seroconversion panels. (DOCX)
