# Supplementary material for: Diagnostic Accuracy of Point-of-Care Tests for Hepatitis C Virus Infection: A Systematic Review and Meta-Analysis
Source: PLoS One. 2015 Mar 27;10(3):e0121450. doi: 10.1371/journal.pone.0121450 (PMC4376712; doi:10.1371/journal.pone.0121450)
Supplement: S1 PRISMA Checklist — (DOC) [file pone.0121450.s001.doc]

|  | | |  |
| --- | --- | --- | --- |
|  |  | **Check list item** | **Reported on page #** |
|  | 1 | Identify the report as a systematic review, meta-analysis, or both. | # See page 1 Title. Title includes both terms systematic review and meta-analysis. |
| **ABSTRACT** | | |  |
| Structured summary | 2 | Provide a structured summary including, as applicable: background; objectives; data sources; study eligibility criteria, participants, and interventions; study appraisal and synthesis methods; results; limitations; conclusions and implications of key findings; systematic review registration number. | # see page 2. Abstract under headings: Background, Methods, Findings and Conclusions. All items identified in the Abstract. |
| **INTRODUCTION** | | |  |
| Rationale | 3 | Describe the rationale for the review in the context of what is already known. | # see introduction, page 3 para 1 to 3. Importance of this study identified. Reference is made of one earlier meta-analysis on the subject and its limitations and reason for doing one more. |
| Objectives | 4 | Provide an explicit statement of questions being addressed with reference to participants, interventions, comparisons, outcomes, and study design (PICOS). | # see introduction page 3 para 2. Three unanswered questions which are most relevant and could be answered by this meta-analysis have been identified. |
| **METHODS** | | |  |
| Protocol and registration | 5 | Indicate if a review protocol exists, if and where it can be accessed (e.g., Web address), and, if available, provide registration information including registration number. | #see page 3 last para under subheading “Protocol”. Protocol has been published and holds a registration number (ID=CRD42014008919). It can be reached at <http://www.crd.york.ac.uk/PROSPERO/display_record.asp>? [ID=CRD42014008919] The protocol is included as supplementary file: Document S2. |
| Eligibility criteria | 6 | Specify study characteristics (e.g., PICOS, length of follow-up) and report characteristics (e.g., years considered, language, publication status) used as criteria for eligibility, giving rationale. | # see page 4 para 3 under “Criteria for study selection”. All defined characteristics included. |
| Information sources | 7 | Describe all information sources (e.g., databases with dates of coverage, contact with study authors to identify additional studies) in the search and date last searched. | # see page 4 para 2 under “Acquisition of Data”. Used “Metasearch engine” to search data. In addition exhaustive search was done to acquire the data. Metasearch can be reached at: <http://mengs1.cs.binghamton.edu/metta/search.action>  Characteristics and advantages of use of Metasearch engine in systematic reviews are identified and given in text as well as in supplementary document S3. |
| Search | 8 | Present full electronic search strategy for at least one database, including any limits used, such that it could be repeated. | # see search strategy under “Acquisition of data” page 4 para 2. Metasearch engine was used and search terms are given. |
| Study selection | 9 | State the process for selecting studies (i.e., screening, eligibility, included in systematic review, and, if applicable, included in the meta-analysis). | # see page 4 para 3 under “Criteria for study selection”. All criteria included. |
| Data collection process | 10 | Describe method of data extraction from reports (e.g., piloted forms, independently, in duplicate) and any processes for obtaining and confirming data from investigators. | # see page 4 first para. Two reviewers made literature search, performed quality assessment of the included studies and extracted data for estimating test accuracy. Any discrepancies were referred to third reviewer. |
| Data items | 11 | List and define all variables for which data were sought (e.g., PICOS, funding sources) and any assumptions and simplifications made. | # see page 4 last para and page 5 first para under “Data Extraction”. Defines all variables in studies and tests. |
| Risk of bias in individual studies | 12 | Describe methods used for assessing risk of bias of individual studies (including specification of whether this was done at the study or outcome level), and how this information is to be used in any data synthesis. | Studies were assessed by using QUADAS-2 and STARD checklists (see page 5 para 2 under quality Assessment). |
| Summary measures | 13 | State the principal summary measures (e.g., risk ratio, difference in means). | # see page 5 para 4 under “Statistical Analysis”. Five measures sensitivity, specificity, positive likelihood ratio, negative likelihood ration and diagnostic Odds ratio were calculated. |
| Synthesis of results | 14 | Describe the methods of handling data and combining results of studies, if done, including measures of consistency (e.g., I^2^) for each meta-analysis. | # see page 5 para 4 under Statistical Analysis. Data were analysed with Meta-Analyst software. Consistency measure (Inconsistency Index I^2^) was calculated and reported. |

Page 1 of 2

|  |  | **Checklist item** | **# reported on page** |
| --- | --- | --- | --- |
| Risk of bias across studies | 15 | Specify any assessment of risk of bias that may affect the cumulative evidence (e.g., publication bias, selective reporting within studies). | Sources of heterogeneity were extensively worked out to assess the heterogeneity between studies. See Page 5 para 4 for details of how heterogeneity was assessed. |
| Additional analyses | 16 | Describe methods of additional analyses (e.g., sensitivity or subgroup analyses, meta-regression), if done, indicating which were pre-specified. | Both subgroup analysis and meta-regression studies were performed. See page 5 para 4. |
| **RESULTS** | | |  |
| Study selection | 17 | Give numbers of studies screened, assessed for eligibility, and included in the review, with reasons for exclusions at each stage, ideally with a flow diagram. | # See Page 5 last para for details under “Literature search and study characteristics”. Also see Fig 1 for details (Flow diagram for study selection). |
| Study characteristics | 18 | For each study, present characteristics for which data were extracted (e.g., study size, PICOS, follow-up period) and provide the citations. | # See Page 5 last para for details under “Literature search and study characteristics”. Also see table 1 for details of included studies. |
| Risk of bias within studies | 19 | Present data on risk of bias of each study and, if available, any outcome level assessment (see item 12). | See page 6 para 6 for description of bias of studies. Details are in QUADAS 2 with STARD checklist (table 3). |
| Results of individual studies | 20 | For all outcomes considered (benefits or harms), present, for each study: (a) simple summary data for each intervention group (b) effect estimates and confidence intervals, ideally with a forest plot. | See page 6 last para along with Forest plot (Fig 2). |
| Synthesis of results | 21 | Present results of each meta-analysis done, including confidence intervals and measures of consistency. | # See page 6 last para and page 7 first para under pooled test accuracy. Also see ROC curve (Fig 3). |
| Risk of bias across studies | 22 | Present results of any assessment of risk of bias across studies (see Item 15). | # See page 7 para 2 under sources of heterogeneity. |
| Additional analysis | 23 | Give results of additional analyses, if done (e.g., sensitivity or subgroup analyses, meta-regression [see Item 16]). | # see page 7 para 3 and 4 under analytical analysis and individual test accuracy. See table 4 and 5 for subgroup results and meta-regression data). |
| **DISCUSSION** | | |  |
| Summary of evidence | 24 | Summarize the main findings including the strength of evidence for each main outcome; consider their relevance to key groups (e.g., healthcare providers, users, and policy makers). | # see page 8 para 4 under Discussion. Six strengths of the study have been identified. See page 10 para defines relevance of these data to policymakers and hospital administrators. |
| Limitations | 25 | Discuss limitations at study and outcome level (e.g., risk of bias), and at review-level (e.g., incomplete retrieval of identified research, reporting bias). | # see page 10 para 3. Limitations of the meta-analysis defined. |
| Conclusions | 26 | Provide a general interpretation of the results in the context of other evidence, and implications for future research. | # see page 10 para 1, 2 and 4. |
| **FUNDING** | | |  |
| Funding | 27 | Describe sources of funding for the systematic review and other support (e.g., supply of data); role of funders for the systematic review. | Defined. No funding for this study. |

***From:*  Moher D, Liberati A, Tetzlaff J, Altman DG, The PRISMA Group (2009). Preferred Reporting Items for Systematic Reviews and Meta-Analyses: The PRISMA**

**Statement. PLoS Med 6(6): e1000097. doi:10.1371/journal.pmed1000097**

**For more information, visit: www.prisma-statement.org.**

Page 2 of 2
