# Supplementary figures and images for: Diagnostic Accuracy of Point-of-Care Tests for Hepatitis C Virus Infection: A Systematic Review and Meta-Analysis
Source: PLoS One. 2015 Mar 27;10(3):e0121450. doi: 10.1371/journal.pone.0121450 (PMC4376712; doi:10.1371/journal.pone.0121450)

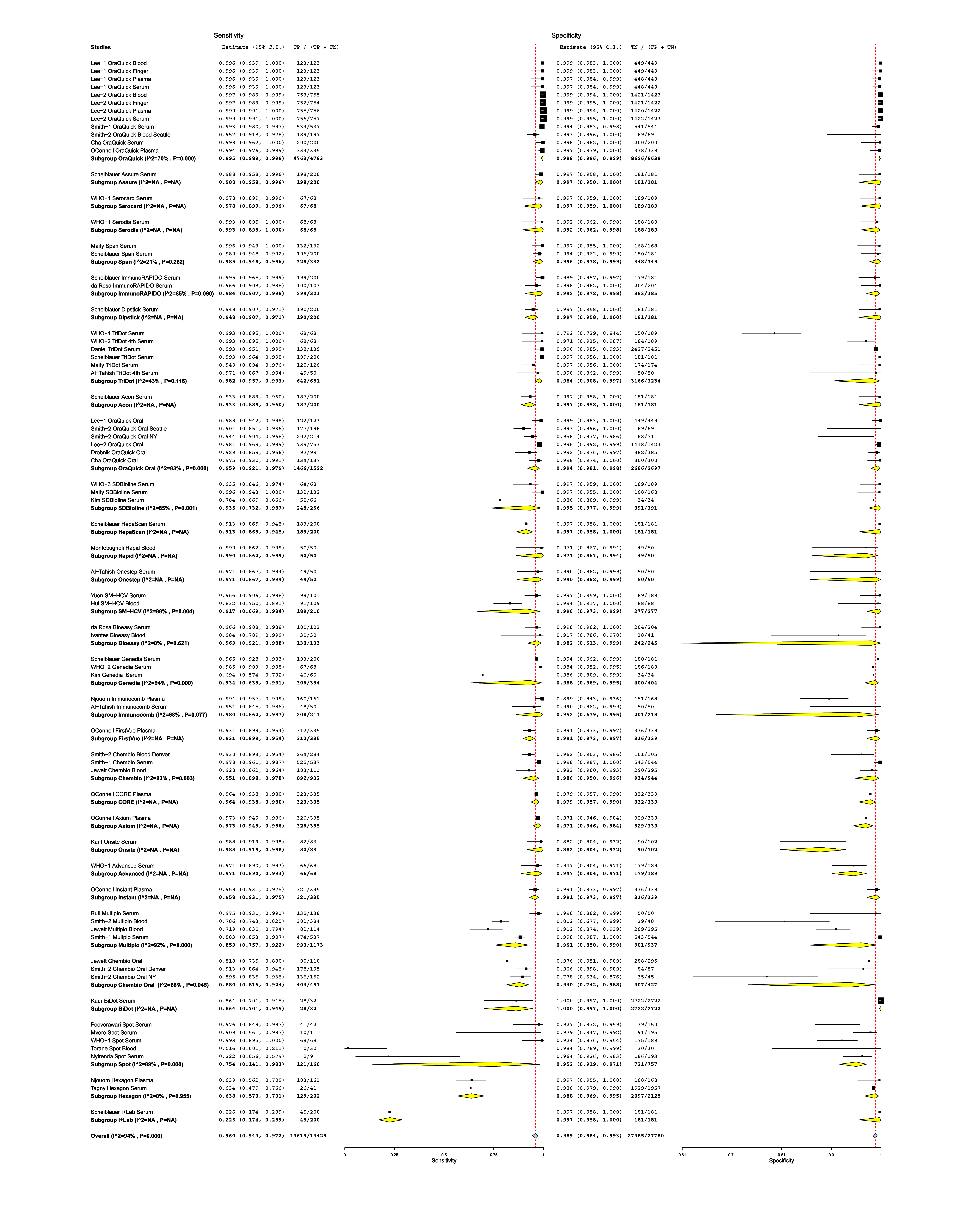

Supplement: S1 Figure — Tests are placed in descending order based on the test estimates. Thirty studies had used 30 test brands and generated 73 data points. Estimates of TriDot and TriDot 4th were similar and have been clubbed together. Estimates of two tests (OraQuick and Chembio) obtained on oral fluid testing are shown separately. Estimates of sensitivity and specificity from each study are shown as solid squares. Solid lines represent the 95% CIs. Squares are proportional to the weights based on the random effect model. Pooled estimates and 95% CIs is denoted by the diamond at the bottom. I^2 and p values represents heterogeneity of studies. (TIF) [file pone.0121450.s002.tif]

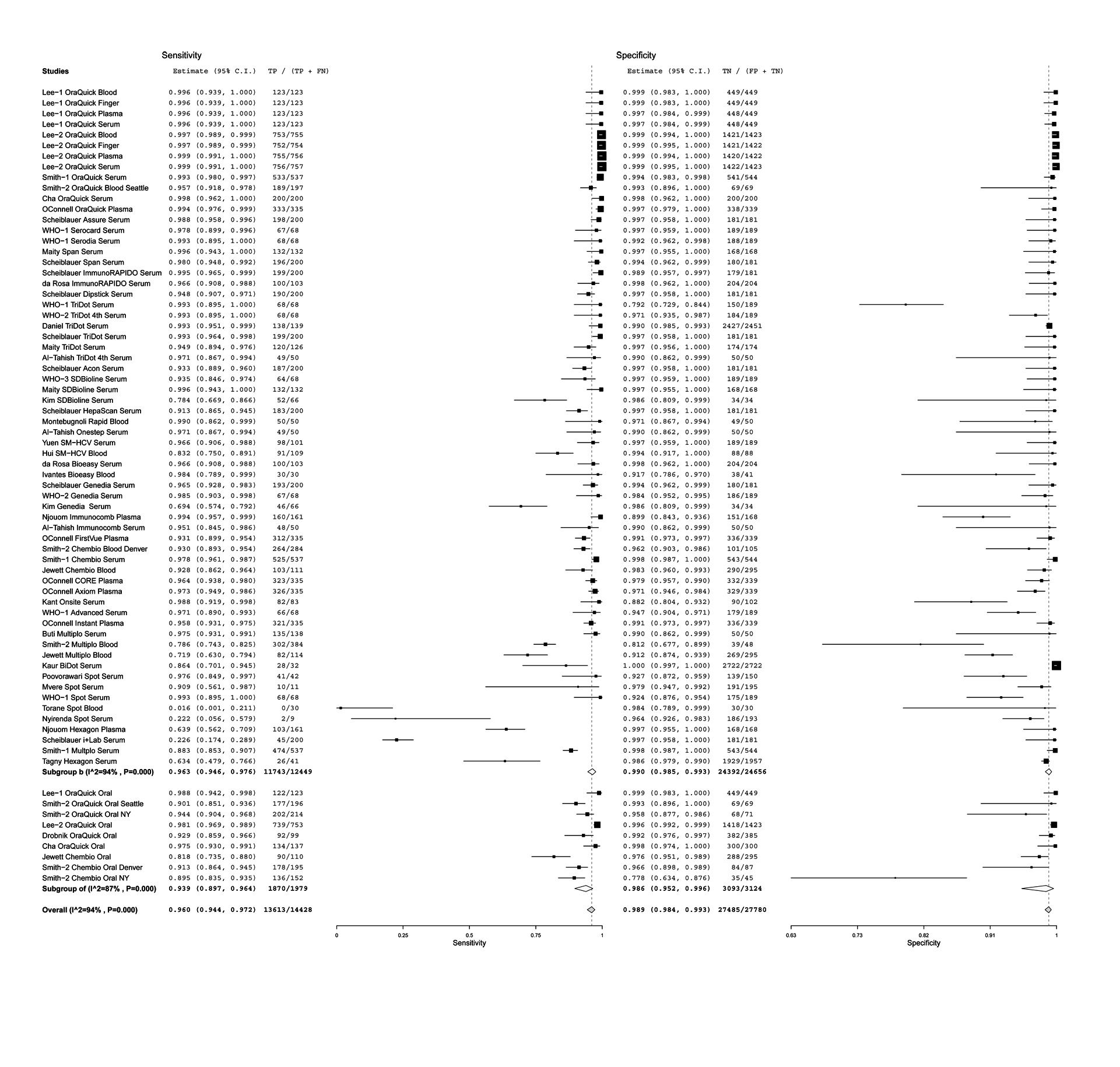

Supplement: S2 Figure — Whole blood, finger stick, plasma, and serum samples had generated 64 data points (b) and oral fluid samples had generated 9 data points (of). Estimates of sensitivity and specificity from each study are shown as solid squares. Solid lines represent the 95% CIs. Squares are proportional to the weights based on the random effect model. Pooled estimates and 95% CIs is denoted by the diamond at the bottom. I^2 and p values represents heterogeneity of studies. (TIF) [file pone.0121450.s003.tif]

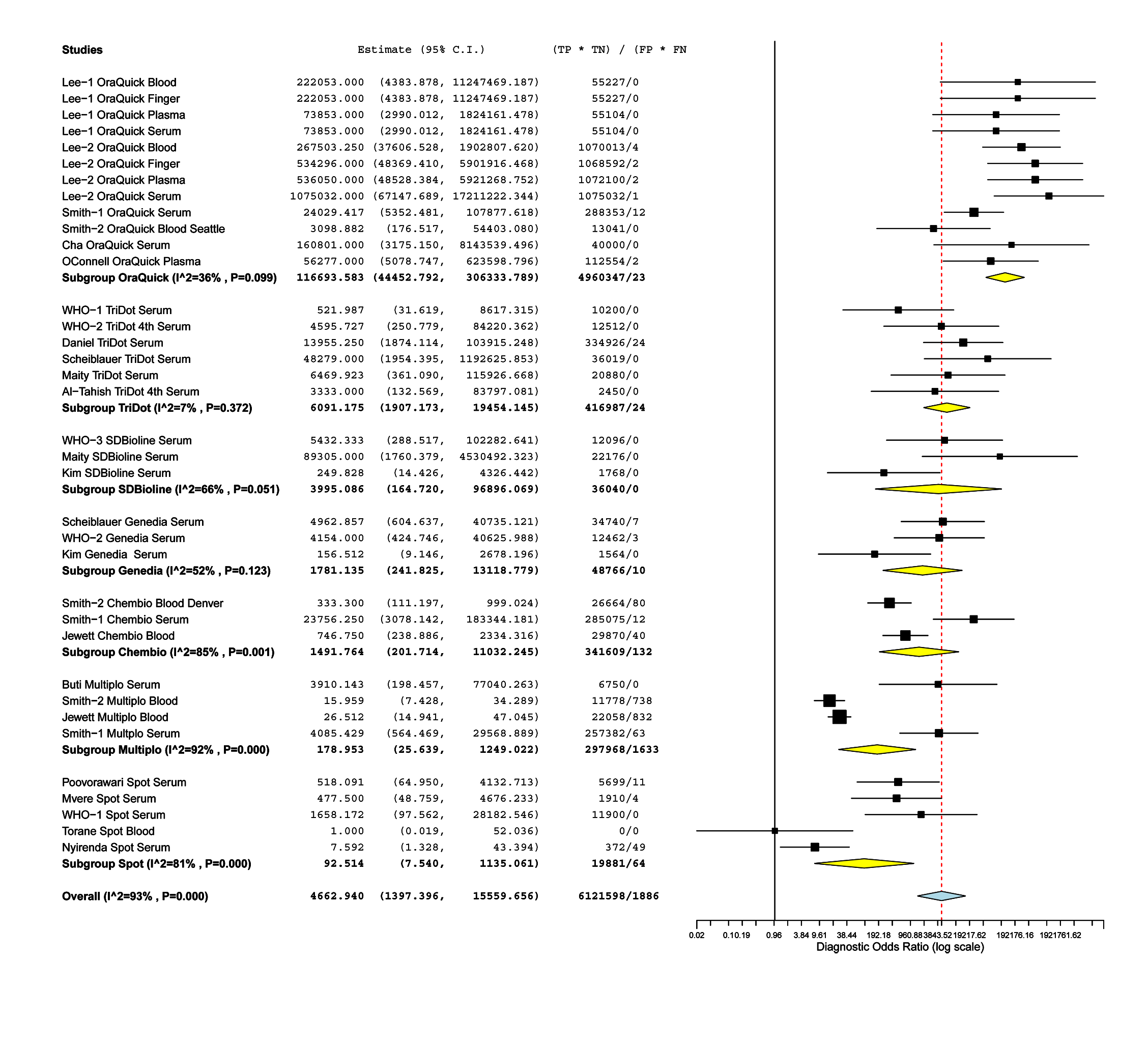

Supplement: S3 Figure — Diagnostic odds ratio from each data point are shown as solid squares. Solid lines represent the 95% CIs. Squares are proportional to the weights based on the random effect model. Pooled estimates and 95% CIs is denoted by the diamond at the bottom. I^2 and p values represents heterogeneity of studies. (TIF) [file pone.0121450.s004.tif]

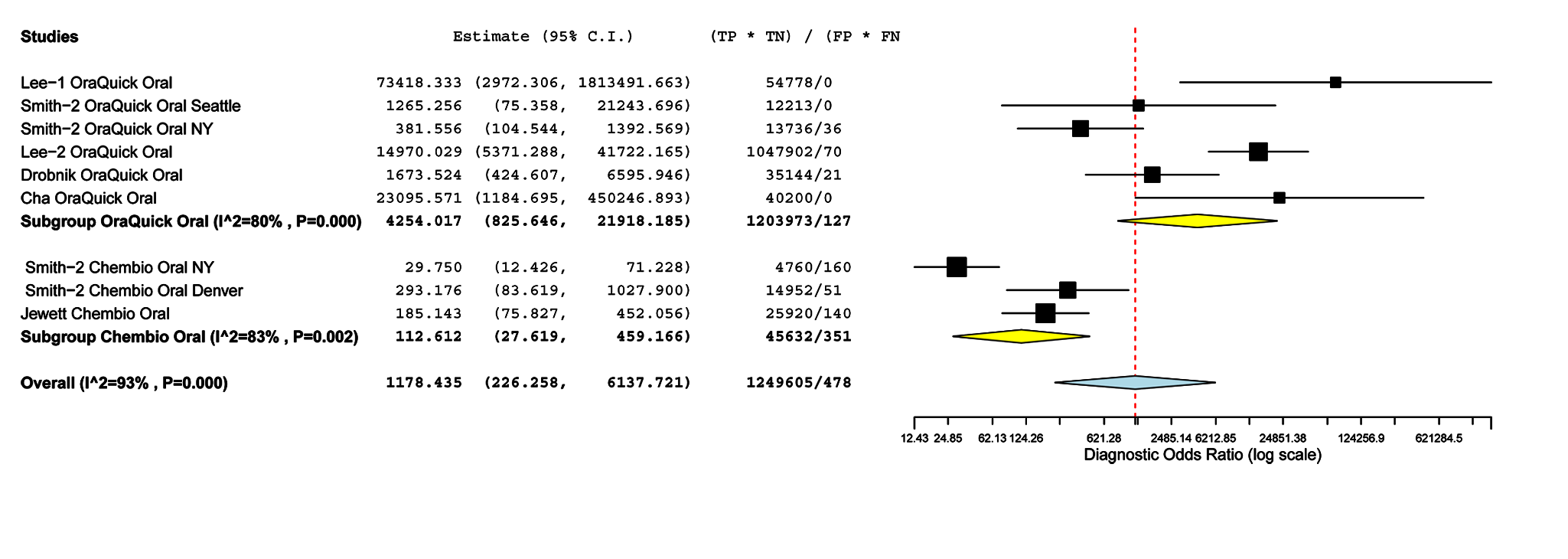

Supplement: S4 Figure — Diagnostic odds ratio from each data point are shown as solid squares. Solid lines represent the 95% CIs. Squares are proportional to the weights based on the random effect model. Pooled estimates and 95% CIs is denoted by the diamond at the bottom. I^2 and p values represents heterogeneity of studies. (TIF) [file pone.0121450.s005.tif]

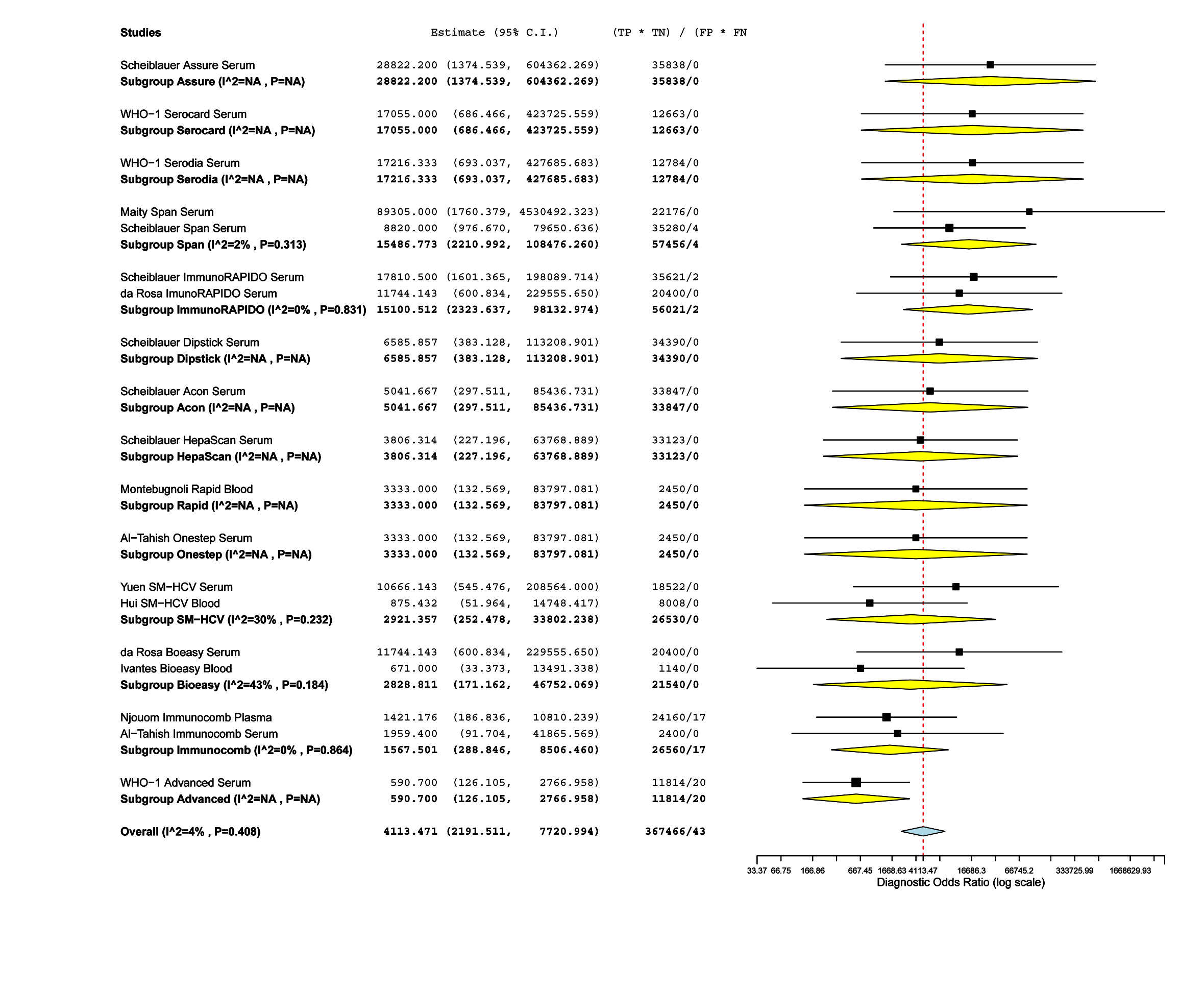

Supplement: S5 Figure — Diagnostic odds ratio each data point are shown as solid squares. Solid lines represent the 95% CIs. Squares are proportional to the weights based on the random effect model. Pooled estimates and 95% CIs is denoted by the diamond at the bottom. I^2 and p values represents heterogeneity of studies. (TIF) [file pone.0121450.s006.tif]
