# Supplementary material for: Diagnostic Accuracy of Point-of-Care Tests for Hepatitis C Virus Infection: A Systematic Review and Meta-Analysis
Source: PLoS One. 2015 Mar 27;10(3):e0121450. doi: 10.1371/journal.pone.0121450 (PMC4376712; doi:10.1371/journal.pone.0121450)
Supplement: S1 File — Systematic review methodology & definitions of relevant accuracy estimates. (DOCX) [file pone.0121450.s007.docx]

**File S1**

**Methodology Document.**

**Systematic Review Methodology & Definitions of Relevant Accuracy Estimates.**

**(I) LITERATURE SEARCH:** Literature search was done with the help of a metasearch engine “Mettā” which can be accessed at

<http://mengs1.cs.binghamton.edu/metta/search.action>. Mettā is a query interface for retrieving articles from five leading medical databases: PubMed, EMBASE, CINAHL, PsycINFO, and the Cochrane Central Register of Controlled Trials. Metta is a multitool pipeline that assists systematic reviewers in retrieving, filtering and assessing publications. The articles can be exported in XML format for further processing by a series of other computer-based tools, or exported in BibTex format for users who employ reference manager software.

Reference: Smalheiser NR, Lin C, Jia L, Jiang Y, Cohen AM, Yu C, Davis JM, Adams CE, McDonagh MS, Meng W. Design and implementation of metta, a metasearch engine for biomedical literature retrieval intended for systematic reviewers *Health Information Science and Systems* 2014, 2:1 doi:10.1186/2047-2501-2-1.

(II) **REPORTING AND ANALYZING INCONCLUSIVE TEST RESULTS:** Diagnostic tests results often include a subset of results that are relatively uninformative and lead to inconclusive diagnostic outcome. An unfortunately common approach to dealing with this type of valid but inconclusive result is to exclude them completely from all analyses. There are few instances where this can be justified, and this approach can lead to overstated summary statistics and promotion of suboptimal test strategies. The STARD (STAndards for the Reporting of Diagnostic accuracy studies) statement—a reporting guideline encouraging high quality reporting of diagnostic accuracy studies—recommends that authors “report how indeterminate results, missing responses and outliers of the index tests are handled.”

Reporting and analysis of inconclusive test results have been relatively neglected in diagnostic accuracy studies. We encourage researchers and clinicians to clearly report all inconclusive results, broken down by the reference standard when possible. Complete transparency regarding the handling of inconclusive results in the analysis phase is essential for the reader to understand how key summary statistics have been derived. A well reported diagnostic accuracy study will allow readers to fully understand if and how inconclusive results were incorporated into analyses and provide them with sufficient information to recalculate key statistics if they disagree with the approach adopted by the author.

We believe that these valid inconclusive results should be grouped in to either the positive or negative results, depending on how these patients would be treated in the clinical context. For the present meta-analysis all inconclusive results would be considered as either false positive or false negative and we approached this as follows:

We grouped results from each study in to 3x2 classification matrix as shown below and grouped intermediate, indeterminate, and uninterpretable results in to a row of “uncertain” or “inconclusive” results. The inconclusive results were clubbed with false positive or false negative results to constitute a 2x2 classification matrix for statistical calculations.

| **3×2 classification table, with allowance for reporting of valid inconclusive test results** | | |
| --- | --- | --- |
|  | Disease State (Reference test results) | |
| Index Test Results | Present (Positive) | Absent (Negative) |
| Positive | True positive | False positive |
| Valid Inconclusive | Disease present but results inconclusive (Include as false positive results) | Disease absent  but results inconclusive (Include as false negative results) |
| Negative | False negative | True negative |

Reference: Shinkins B, Thompson M, Mallett S, Perera R. Diagnostic accuracy studies: how to report and analyse inconclusive test results. *BMJ* 2013;346:f2778. doi: 10.1136/bmj.f2778

**(III) DATA TABULATION:** We defined Anti-HCV positive as those with Disease and Anti-HCV negative as those without disease as defined by reference test. Test outcome (index test) was reported as positive and negative.

A 2x2 table defining disease status and test results was made as follows:

| Index test  outcome | Reference Test Results | | Total |
| --- | --- | --- | --- |
|  | Anti-HCV positive | Anti-HCV negative |  |
| Anti-HCV positive | True positive (**a**) | False positive (**b**) | Index test  positives (**a + b**) |
| Anti-HCV negative | False negative (**c**) | True negative (**d**) | Index test  negatives (**c + d**) |
| Total | Reference test positives (**a + c**) | Reference test negatives (**b + d**) | N (**a + b + c + d**) |

**(IV) SENSITIVITY & SPECIFICITY:** Sensitivity of a test is defined as the probability that the index test result will be positive in a diseased case.

**Sensitivity= True positive ÷ [True positive + False negative] =** **(a) ÷ (a + c)**.

Specificity of a test is defined as the probability that the index test result will be negative in a non‐diseased case.

**Specificity= True negative ÷ [False positive + True negative] =** (**d**) **÷** (**b + d**).

Both Sensitivity, & Specificity can be expressed as proportions or percentages.

**(V) LIKELIHOOD RATIOS:** Likelihood ratio (LR) can be used to update the pre‐test probability of disease using Bayes’ theorem, once the test result is known. The updated probability is referred to as the post‐test probability. For a test that is informative, the post‐test probability should be higher than the pre‐test probability if the test result is positive, whereas the post‐test probability should be lower than the pre‐test probability if the test result is negative.

Positive LR describes how many times more likely positive index test results were in the diseased group compared to the non‐diseased group. The positive LR, which should be greater than 1 if the test informative.

**Positive LR= Sensitivity ÷ (1−Specificty) =** **[(a) ÷ (a + c)] ÷ [1-{(d) ÷ (b + d)}]**.

Negative LR describes how many times less likely negative index test results were in the diseased group compared to the non‐diseased group. Negative LR should be less than 1 if the test is informative.

**Negative LR = (1–Sensitivity) ÷ Specificity = [1- {(a) ÷ (a + c)}] ÷ [(d) ÷ (b + d)].**

Positive and negative LR describe the discriminatory properties of a positive and negative test and results are interpreted as follows:

| **Likelihood ratio (LR)** | | **Test interpretation** |
| --- | --- | --- |
| **LR+** | **LR-** |  |
| >10 | <0.1 | Conclusive evidence |
| 5-10 | 0.1-0.2 | Strong diagnostic evidence |
| 2-5 | 0.2-0.5 | Weak diagnostic evidence |
| 1-2 | 0.5-1.0 | Negligible evidence |

**(VI) DIAGNOSTIC ODDS RATIOS:** Diagnostic odds ratio (DOR) summarizes the diagnostic accuracy of the index test as a single number that describes how many times higher the odds are of obtaining a test positive result in a diseased rather than a non‐diseased person. The fact that it summarizes test accuracy in a single number makes it easy to use this measure for meta‐analysis but expressing accuracy in terms of ratios of odds means the measure has little direct clinical relevance, and it is rarely used as a summary statistic in primary studies. In fact, the clinician is usually interested in the sum of the number of false negative and false positive results whereas the DOR reflects their product. The DOR does, however, remain an important element in meta‐analytic model building.

**DOR = [(Sensitivity × Specificity)] ÷ [(1–Sensitivity) × (1–Specificity)]**

**or**

**DOR= [Sensitivity ÷ (1- Sensitivity)] ÷ [(1- Specificity) ÷ Specificity]**

**or**

**DOR=Positive LR ÷ Negative LR = (ad) ÷ (bc)**

The natural logarithm of the Odds ratio is designated as D [D=logit (TPP)-logit (FPP) and S=logit (TPP) +Logit (FPP).

After the data from each primary study have been summarized by calculating 2 quantities (Di & Si for the ith study) analysis fit a simple linear regression model using D as dependent variable and S as the predictive variable as: D= alpha +beta S. TPP=True Positive Proportion or sensitivity, FPP=False Positive Proportion or Specificity.

**(VII) IDENTIFY AND MEASURE HETEROGENEITY:** Heterogeneity was identified and assessed as follows:

i) Overlap in the confidence intervals of individual studies. Poor overlap depicted statistical heterogeneity,

ii. Chi-squared (χ^2^, or Chi^2^) test for heterogeneity with P value. A large χ^2^ value with P <0.10 (rather than conventional 0.05) suggested heterogeneity,

iii) Calculating I^^2^ for heterogeneity: I^^2^ is calculated as follows^:.^


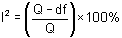


Where Q=Chi square value for heterogeneity; df=degree of freedom

A rough guide to interpretation is as follows:

| **I^2 value** | **Magnitude of Heterogeneity** |
| --- | --- |
| <25% | Might not be important |
| >25% to 50% | May represent moderate heterogeneity |
| >50% to 75% | May represent substantial heterogeneity |
| >75%% | Considerable heterogeneity |
| The importance of the observed value of I^2^ depends on (i) magnitude and direction of effects and (ii) strength of evidence for heterogeneity (e.g. P value from the chi-squared test, or a confidence interval for I^2^ ). | |

**(VIII) HOW TO READ RECEIVER OPERATING CHARACTERISTIC (ROC) PLOT CURVES:** The ROC curve of a test is the graph of the values of sensitivity and specificity that are obtained by varying the positivity threshold across all possible values. The graph plots sensitivity (true positive rate) against 1–specificity (false‐positive rate). The curve for any test moves from the point where sensitivity and 1–specificity are both 1 (the upper right corner) which is achieved for a threshold at the lower end of its range (classifying all participants as test positive, so there are no false negatives but many false positives) to a point where sensitivity and 1‐specificity are both zero (the lower left corner) which is achieved when the threshold moves to the upper end of its range (and all participants are classified as test negative, giving no false positives but many false negatives). The shape of the curve between these two fixed points depends on the discriminatory ability of the test.

ROC curve is estimated from a finite sample of test results and hence will not necessarily be a smooth curve. The horizontal axis for each ROC plot is labelled in terms of specificity decreasing from 1.0 to 0.0. This style of labelling is (1‐specificity ranging from 0.0 to 1.0).

The position of the ROC curve depends on the degree of overlap of the distributions of the test measurement in diseased and non‐diseased. Where a test clearly discriminates between diseased and non‐diseased such that there is no or little overlap of distributions, the ROC curve will indicate that high sensitivity is achieved with a high specificity, that is the curve approaches the upper left hand corner of the graph where sensitivity is 1 and specificity is 1. If the distributions of test results in diseased and non‐diseased coincide, the test would be completely uninformative and its ROC curve would be the upward diagonal of the square.

The ROC curves may be symmetrical about the sensitivity=specificity line (the downward diagonal of the square) or not symmetrical. Asymmetrical curves typically occur when the distribution of the test measurement in those with disease has more or less variability than the distribution in nondiseased people. Increased variability might occur, for example, where disease may cause a biomarker both to rise and become more erratic; reduced variability might occur where disease may lower biomarker values to a bounding level such as a lower level of detection.

**(IX) COUPLED FOREST PLOTS:** Forest plots for diagnostic test accuracy report the number of true positives and false negatives in diseased and true negatives and false positives in non‐diseased participants in each study, and the estimated sensitivity and specificity, together with confidence intervals. The plots are known as coupled forest plots as they contain two graphical sections: one depicting sensitivity, and one specificity. The order of the studies can be sorted, often they are presented sorted by values of sensitivity, or grouped by test type or covariate values. Whilst it is possible to observe heterogeneity in sensitivity and specificity individually on such plots, it is not as easy to visualize whether there are threshold‐like relationships. Summary statistics computed from meta‐analyses can be added to coupled forest plots.
