# Supplementary material for: Diagnostic Accuracy of Point-of-Care Tests for Hepatitis C Virus Infection: A Systematic Review and Meta-Analysis
Source: PLoS One. 2015 Mar 27;10(3):e0121450. doi: 10.1371/journal.pone.0121450 (PMC4376712; doi:10.1371/journal.pone.0121450)
Supplement: S1 Protocol — (PDF) [file pone.0121450.s008.pdf]

PROTOCOL FOR SYSTEMATIC REVIEW AND META-ANALYSIS

TITLE

ACCURACY OF RAPID AND POINT-OF-CARE DIAGNOSTIC TESTS FOR HEPATITIS C: A SYSTEMATIC REVIEW AND META-ANALYSIS

REVIEW PURPOSE

The purpose of the present study was to evaluate the diagnostic accuracy of rapid point-of-care tests for Hepatitis C. For this we proposed to conduct a systematic review and meta-analysis on studies which evaluated accuracy of rapid diagnostic tests (RDTs) and point-of-care tests (POCTs) for Hepatitis C.

REVIEW TEAM & AFFLIATION:

1) Khuroo Mehnaaz Sultan, MD (Pathology), Assistant Professor Pathology, Government Medical College Srinagar, Kashmir, J&K, India, 190010; E-mail: mkhuroo@yahoo.com;
Phone: +91-194-2490442, Cell phone: +91-9906680006
2) Khuroo Naira Sultan, MBBS, Fellowship Radiology (KFSHRC Riyadh) Consultant Radio diagnosis, Digestive Diseases Centre, Dr Khuroo Medical Clinic, Srinagar, Kashmir, J&K, India, 190010; E-mail: naira_sultan@yahoo.com Phone: +91-194-2492398, cell phone: +91-9906633996
3) Khuroo Mohammad Sultan, MD, DM, FRCP (Edinburgh), FACP, MACP Director, Digestive Diseases Centre, Dr. Khuroo's Medical Clinic, Srinagar, Kashmir, J&K, India, 190010; E-mail: khuroo@yahoo.com Phone: +91-194-2491190, cell phone: +91-9906591044

CORRESPONDENCE TO:

Khuroo Mohammad Sultan, MD, DM, FRCP (Edinburgh), FACP, MACP Director, Digestive Diseases Centre, Dr. Khuroo's Medical Clinic, Srinagar, Kashmir, J&K, India, 190010; E-mail: khuroo@yahoo.com Phone: +91-194-2491190, cell phone: +91-9906591044.

Abbreviations used in this protocol study:
HCV=Hepatitis C Virus; Anti-HCV= Antibody to hepatitis C virus; POCTs=Point-of-Care Tests; RDTs=Rapid Diagnostic Tests; PRISMA=Preferred Reporting Items for Systematic Reviews and Meta-analysis; QUADAS=Quality Assessment of Diagnostic Accuracy of Studies; STARD=Standard for Reporting Diagnostic Accuracy Studies; SROC=Summary Receiver Operating Characteristic; EIA=Enzyme Immunoassay; MEIA=Micro particle enzyme immunoassay; ELISA=Enzyme Linked Immunosorbant Assay; PCR=Polymerase chain Reaction; LR=Likelihood Ratio; DOR=Diagnostic Odds Ratio; HSROC=Hierarchical Summary Receiver Operating Characteristic.

Funding Source
Our study shall be funded by the "Dr. Khuroo's Medical Trust", a non-profit organization to promote research and help poor patients for medical treatment. The funding source had no role in the conception, design or conduct of this review.

Conflict of interest
None of the authors have any conflict of interest as regards to this proposed study.

Specific Team Contributions:
Conception and design: Khuroo Mehnaaz Sultan; Data abstraction: Khuroo Mehnaaz Sultan & Khuroo Naira Sultan; Data analysis and interpretation: Khuroo Mohammad Sultan and Khuroo Mehnaaz Sultan; writing of the manuscript: Khuroo Mohammad Sultan and Khuroo Mehnaaz Sultan.

BROAD GUIDELINES FOR THIS SYSTEMATIC REVIEW AND META-ANALYSIS

**Item**	**Specifications**
Type of meta-analysis	**Diagnostic Test Accuracy** (DTA)
Basis of reporting	**PRISMA** (Preferred Reporting Items for Systematic Reviews and Meta-analysis) guidelines.
Search Engines	**MEDLINE** (via PUBMED), **EMBASE** (via OVID), **BIOSIS** and **Web of Science** (1980 to December 2013).
MESH Terms employed for search	**"Hepatitis C"** OR **"Hepatitis C Antibodies"** OR **"Hepatitis C Virus"** OR **"Hepatitis C Antigens"** AND **"Point-of-Care Systems"** OR **"rapid test"** OR **"diagnostics"** AND **"Sensitivity and Specificity"** OR **"diagnostic accuracy"** OR **"validity".**
Index Test	**Rapid Diagnostic Tests** (RDTs); **Point-of-Care Tests** (POCTs)
Reference Test	**ELISA, EIA, MEIA**
Quality Assessment of Studies	**QUADAS-2** (Quality Assessment of Diagnostic Accuracy Studies) tool
Scoring of the Studies	the **STARD** (Standards for the Reporting of Diagnostic Accuracy Studies) checklists
Software for Statistical Analysis	**Meta-Analyst** (Tufts Medical Centre, Boston, MA)

Metrics used	**Sensitivity** & **specificity**, **Positive** & **Negative likelihood ratio** [LR]) and **Diagnostic odd ratios** [DORs] along with 95 percent confidence intervals (CIs)
Data Representation	**Forest Plots; SROC**
Statistical Model	**Bivariate model**; **Random effects model**
Subgroup Analysis	Subgroups estimates shall be compared in **Meta-regression** model
Evaluation of Heterogeneity	**Q test** statistic (Chi square value with p values) and **$I^{\wedge 2}$** values. Potential sources compared in **Meta-regression** model
Questions to be answered from the study?	**Accuracy of individual tests**, **Analytical sensitivity of the tests**, **Comparative efficacy of the different tests**, **Heterogeneity** within and between studies and their potential sources, **Applicability** of these tests in different scenarios of HBV evaluation.

BACKGROUND

Hepatitis C is a global health problem (1). An estimated 2 to 3% of the world population is chronically infected with hepatitis C virus (HCV). This amounts to an estimated 130-170 million infected persons worldwide (2). Chronic hepatitis C is associated with significant morbidity and mortality. HCV contributes to 27% of cirrhosis and 25% of hepatocellular carcinoma and causes more than 350 000 deaths each year (3). HCV infection prevalence varies widely throughout the world, even among neighboring countries and in geographic regions within the same country. The prevalence of HCV in the United States, Australia and most countries in Western Europe is less than 2%. HCV infection rates are higher (≥3%) in many countries in Eastern Europe, Latin America, the Middle East, Africa and South Asia. Chronic HCV infection is highly endemic in Egypt (≥10%), many regions of Pakistan and adjoining regions in Western India (≥6%) (4). Another issue which is of importance to the epidemiology of hepatitis C is its relationship with HIV infection. Worldwide up to 30% of the 33 million persons infected with HIV are also infected with HCV. HIV/HCV co-infections also have a varied geographical distribution (5-7). These co-infections are common in sub-Saharan Africa and are becoming common in developed countries where HIV is becoming an increasing problem in men who have sex with men. HIV/HCV co-infection are associated with accelerated progression of liver disease and higher mortality. Mode of spread of HCV in developed countries is mainly through injection drug use. Blood and blood products in these countries is routinely screened for HCV by sensitive methods and there are measures in place to facilitate infection control and safe injection practices. In contrast, unsafe injections in healthcare settings are leading cause of HCV transmission in developing countries. Recipients of blood and blood products also are at risk of infection as up to 20% of such products are not screened for hepatitis viruses in these countries. Also paid or coerced donors, a common occurrence in developing countries are more likely to transmit HCV transmission. Some distinctive risk factors play a part in HCV transmission in some regions of the World. Reuse of syringes during a schistosomiasis eradication program in the 1960 and 1970's attributed to high HCV prevalence in Egypt. Following this HCV spread was purported by widespread use of unsafe injection practice, poor infection control in hospitals and widespread use of unscreened blood for transfusions. In some countries like Japan and Korea, high prevalence of HCV is seen in elderly people with sharp decrease in young generation. This epidemiological pattern along with a disproportionate burden of HCC and liver cirrhosis relative to overall prevalence suggest a high prevalence of HCV infection among persons infected in the distant past. HCV infection in people born between 1945-1965 (Baby Boomers) account for three-fourth of all HCV infections in the United States and Western Europe. One in 30 baby boomers has been infected with hepatitis C, and most have no clue that they are infected. In addition to above around 1 million persons become permanent legal residents in the United States, and many more undocumented persons enter the country. Large number of these persons are from countries where HCV infection is endemic. Of the 40 million foreign-born persons living in the United States, nearly 20 million are from Latin America, where rates of HCV infection approach 3% in some countries. Most of the remainder of immigrants come from Asia, Europe, and Africa serve as countries of origin for remaining immigrants (1-4).

In view of the above, screening of HCV infection in many high risk epidemiologic settings is mandatory. In addition testing of blood and blood products is essential to prevent HCV infection to recipients. Conventionally enzyme immunoassay (EIA) to detect antibody to HCV is the serologic hallmark of hepatitis C infection (8). Nucleic acid testing for HCV RNA and HCV genotype are needed after HCV infection is established on EIA. These tests require high facility cost, sophisticated equipment, trained technicians, continuous supply of electricity, and are unsuitable for use in poor resource endemic regions. Rapid Point-of-care testing offers significant advantages. These are divided in to: i) Point-of-Care tests (POCTs) which need no sample processing, are robust at room temperature and have long shelf-life (>6 months) and ii) Rapid diagnostic tests (RDTs) which require sample processing, storage at 0-4^0 C and have short shelf life (9-11).

Since 1990s, several RDTs and POCTs that primarily use serum, plasma, whole blood and oral fluid to test for anti-HCV have been developed. Manufacturers claim high clinical and analytical sensitivity of these tests. Based on the claims, these tests are widely used in developing countries in many settings including blood banks. However, several vital questions about their use remain unanswered which include: (i) accuracy of individual tests, (ii) comparative efficacy of the different tests, and (iii) applicability of these tests in different scenarios namely population surveys, screening of blood donors, diagnosis of hepatitis C etc.

A recent meta-analysis on accuracy of rapid and point-of-care diagnostic tests for hepatitis C attempted to address the above mentioned questions (12). However, this study had many limitations. Authors did not compare the subgroup estimates in the statistical meta-regression model and thus the interpretation of the meta-analysis were faulty. Analytical sensitivity of the tests based on low titer and sero-conversion panels were not assessed, which affected the conclusions made on the accuracy of the tests under consideration. Authors did not include the evaluation of heterogeneity (differences in reported estimates among studies) and its potential sources, an important component in meta-analysis studies (13). We believe recommendations on use of rapid point-of-care tests can have far reaching effects on the healthcare in developing countries. For example recommending tests with low analytical sensitivity in blood banks can pose a serious threat to recipients as infected otherwise healthy donors often have low titer HCV viremia. Keeping the above in consideration, we conducted another systematic review and meta-analysis of studies pertaining to diagnostic accuracy and applicability of RDTs and POCTs for HCV.

METHODS

Two reviewers shall conduct literature search, quality assessment of the included studies and data extraction for estimating test accuracy (14). Any discrepancies shall be referred to third reviewer. We shall follow **PRISMA** (Preferred Reporting Items for Systematic Reviews and Meta-analysis) guidelines for conducting and reporting on this meta-analysis (15).

Acquisition of Data

The primary search shall be made in MEDLINE (via PUBMED), EMBASE (via OVID), BIOSIS and Web of Science (1980 to December 2013). MeSH terms used for key and text word searching shall be **"Hepatitis C"** OR **"Hepatitis C Antibodies"** OR **"Hepatitis C Virus"** OR **"Hepatitis C Antigens"** AND **"Point-of-Care Systems"** OR **"rapid test"** OR **"diagnostics"** AND **"Sensitivity and Specificity"** OR **"diagnostic accuracy"** OR **"validity".** Bibliographic for the relevant citations and reviews shall be manually searched for relevant citations and experts in the field were contacted to ensure that search strategy is complete. Titles and abstracts of all the above articles identified in the primary search shall be evaluated and a list of potential eligible studies identified. These studies shall be considered for full-text review. Studies which fulfil the criteria for selection shall be included in the systematic review and meta-analysis.

Criteria for Study Inclusion

Following studies shall be included in the meta-analysis:

i. Studies which employed RDTs or POCTs for detection of Anti-HCV (Index test) and compared the results with a reference test and reported results to recreate the 2X2 diagnostic table for estimating test accuracy.
ii. Studies conducted in adults (age>18 years).
iii. Studies published both as abstracts and full-text articles.
iv. Studies using all study-designs, conducted in any study settings (laboratory or field- based) and regardless of sample size, study location, language of publication, and country of origin of test.

Following studies shall be excluded:

i. Studies which deal with accuracy of laboratory-based tests,
ii. Studies with data unable to recreate 2x2 diagnostic table,
iii. reports from the manufacturer and package inserts which are subjected to overt conflict of interest,
iv. Duplicate reports.

Data Extraction

Each study shall be subjected to following search: study author, year of publication, location of study, index test (one or more), reference standard, study design, source of sera, sample size, characteristics of the population employed for sera collection, cross reactive sera included in panel and analytical sera included for evaluating test sensitivity. Detailed information about the index test shall be extracted from the studies which included: name of the test, country of origin and name of the manufacturer, time taken to read results, specimen (serum, plasma, blood or oral fluid) needed for test, volume of the sample (µl) needed to test, storage

conditions for maintaining test kit, special equipment if any needed to perform the test, shelf life of the test kit and scope of the test utility (**RDTs** or **POCTs**). For purposes of data synthesis we shall extract raw cell numbers namely true positives, false negatives, false negatives and true negatives for each test run.

Quality Assessment

Quality assessment of the studies using **QUADAS-2** (Quality Assessment of Diagnostic Accuracy Studies) tool (16) and the **STARD** (Standards for the Reporting of Diagnostic Accuracy Studies) checklists (17) shall be conducted. QUADAS-2 sheet shall be completed by following stepwise guidelines to judge risk of bias (4 domains) and concerns about applicability (3 domains) for each study. STARD checklist consists of 25 questions and each question shall be weighted equally (yes=1, No=0) and total score for each study calculated.

Statistical Analysis and Data Synthesis

While analyzing data, we shall address following questions (i)

i. Accuracy of individual tests,
ii. Analytical sensitivity of the tests,
iii. Comparative efficacy of the different tests,
iv. Heterogeneity within and between studies and their potential sources,
v. Applicability of these tests in different scenarios of HCV evaluation.

To do so following algorithm shall be followed:

Calculation of metrics: For estimates of accuracy we shall use Bivariate model to calculate sensitivity & specificity, positive & negative likelihood ratio [LR]) and Diagnostic odd ratios [DORs] along with 95 percent confidence intervals (CIs) (18). These measures shall be pooled using the random effects model (19). In addition summary receiver operating characteristics (SROC) curve plots shall be obtained (20).

Analytical Sensitivity: Analytical sensitivity of the tests shall be evaluated by analyzing the results of the tests against low titer sera and sero-conversion panels. We shall determine the lowest HCV concentration which shall be picked up by various tests and compare this with what is claimed by the manufacturers.

Subgroup analysis: For further analysis, we shall divide data in to subgroups based on: (i) scope of test utility (RDTs vs. POCTs), (ii) Specimen used to test (blood, plasma, serum or oral fluid, (iii) source of sera (blood banks, hospital/clinic, HIV clinics with included cross reactive sera), (vi) location where test was conducted (developed versus developing countries), (v) tests with sufficient data points will be pooled and the pooled estimates compared with pooled estimates of remaining tests. We shall compare summary estimates of diagnostic accuracy within subgroups to make relevant conclusions.

Heterogeneity: Heterogeneity (differences in reported estimates among studies) shall be evaluated by a Q test statistic (Chi square value with p values) and $I\^{2}$ values (11). Three potential sources of heterogeneity (design of studies; study quality and year of publication) shall be evaluated in the meta-regression model. (19, 20)

We shall use software Meta-Analyst (Tufts Medical Centre, Boston, MA) for all statistical analysis. (21)

DATA TABULATION, CALCULATION OF ESTIMATES, DATA REPRESENTATION, AND EVALUATION OF HETEROGENEITY AND SUBGROUP ANALYSIS

DATA TABULATION

We defined Anti-HCV positive as those with Disease and Anti-HCV negative as those without disease as defined by reference test. Test outcome (index test) was reported as positive and negative.

A 2x2 table defining disease status and test results was made as follows:

Index test outcome	Reference Test Results		Total
	Anti-HCV positive	Anti-HCV negative	
Anti-HCV positive	True positive (**a**)	False positive (**b**)	Index test positives (**a + b**)
Anti-HCV negative	False negative (**c**)	True negative (**d**)	Index test negatives (**c + d**)
Total	Reference test positives (**a + c**)	Reference test negatives (**b + d**)	N (**a + b + c + d**)

Sensitivity & Specificity

Sensitivity of a test is defined as the probability that the index test result will be positive in a diseased case.

Sensitivity= True positive ÷ [True positive + False negative] = (a) ÷ (a + c).

Specificity of a test is defined as the probability that the index test result will be negative in a non-diseased case.

Specificity= True negative ÷ [False positive + True negative] = (**d**) ÷ (**b** + **d**).

Both Sensitivity, & Specificity can be expressed as proportions or percentages.

LIKELIHOOD RATIOS

Likelihood ratio (LR) can be used to update the pre-test probability of disease using Bayes' theorem, once the test result is known. The updated probability is referred to as the post-test probability. For a test that is informative, the post-test probability should be higher

than the pre-test probability if the test result is positive, whereas the post-test probability should be lower than the pre-test probability if the test result is negative.

Positive LR describes how many times more likely positive index test results were in the diseased group compared to the non-diseased group. The positive LR, which should be greater than 1 if the test informative.
Positive LR= Sensitivity ÷ (1–Specificty) = [(a) ÷ (a + c)] ÷ [1-{(d) ÷ (b + d)}].

Negative LR describes how many times less likely negative index test results were in the diseased group compared to the non-diseased group. Negative LR should be less than 1 if the test is informative.
Negative LR = (1–Sensitivity) ÷ Specificity = [1- {(a) ÷ (a + c)}] ÷ [(d) ÷ (b + d)].

Positive and negative LR describe the discriminatory properties of a positive and negative test and results are interpreted as follows:

**Likelihood ratio (LR)**		**Test interpretation**
**LR+**	**LR-**	
>10	<0.1	Conclusive evidence
5-10	0.1-0.2	Strong diagnostic evidence
2-5	0.2-0.5	Weak diagnostic evidence
1-2	0.5-1.0	Negligible evidence

DIAGNOSTIC ODDS RATIOS

Diagnostic odds ratio (DOR) summarizes the diagnostic accuracy of the index test as a single number that describes how many times higher the odds are of obtaining a test positive result in a diseased rather than a non-diseased person. The fact that it summarizes test accuracy in a single number makes it easy to use this measure for meta-analysis but expressing accuracy in terms of ratios of odds means the measure has little direct clinical relevance, and it is rarely used as a summary statistic in primary studies. In fact, the clinician is usually interested in the sum of the number of false negative and false positive results whereas the DOR reflects their product. The DOR does, however, remain an important element in meta-analytic model building.

DOR = [(Sensitivity × Specificity)] ÷ [(1–Sensitivity) × (1–Specificity)]

or

DOR= [Sensitivity ÷ (1- Sensitivity)] ÷ [(1- Specificity) ÷ Specificity]

or

DOR=Positive LR ÷ Negative LR = (ad) ÷ (bc)

The natural logarithm of the Odds ratio is designated as D [D=logit (TPP)-logit (FPP) and S=logit (TPP) +Logit (FPP).

After the data from each primary study have been summarized by calculating 2 quantities (Di & Si for the ith study) analysis fit a simple linear regression model using D as dependent variable and S as the predictive variable as: D= alpha +beta S. TPP=True Positive Proportion or sensitivity, FPP=False Positive Proportion or Specificity.

IDENTIFY AND MEASURE HETEROGENEITY

Heterogeneity was identified and assessed as follows:

i) Overlap in the confidence intervals of individual studies. Poor overlap depicted statistical heterogeneity,

ii. Chi-squared (χ^2, or Chi^2) test for heterogeneity with P value. A large χ^2 value with P <0.10 (rather than conventional 0.05) suggested heterogeneity,

iii) Calculating $I^{\wedge 2}$ for heterogeneity: $I^{\wedge 2}$ is calculated as follows:

$$I^2 = \left(\frac{Q - df}{Q}\right) \times 100\%$$

Where Q=Chi square value for heterogeneity; df=degree of freedom

A rough guide to interpretation is as follows:

**I^2 value**	**Magnitude of Heterogeneity**
0% to 40%	Might not be important
30% to 60%	May represent moderate heterogeneity
50% to 90%	May represent substantial heterogeneity
75% to 100%	Considerable heterogeneity

The importance of the observed value of I^2 depends on (i) magnitude and direction of effects and (ii) strength of evidence for heterogeneity (e.g. P value from the chi-squared test, or a confidence interval for I^2).

HOW TO READ RECEIVER OPERATING CHARACTERISTIC (ROC) PLOT CURVES

The ROC curve of a test is the graph of the values of sensitivity and specificity that are obtained by varying the positivity threshold across all possible values. The graph plots sensitivity (true positive rate) against 1–specificity (false-positive rate). The curve for any test moves from the point where sensitivity and 1–specificity are both 1 (the upper right corner) which is achieved for a threshold at the lower end of its range (classifying all participants as test positive, so there are no false negatives but many false positives) to a point where sensitivity and 1-specificity are both zero (the lower left corner) which is achieved when the threshold moves to the upper end of its range (and all participants are classified as test negative, giving no false positives but many false negatives). The shape of the curve between these two fixed points depends on the discriminatory ability of the test.

ROC curve is estimated from a finite sample of test results and hence will not necessarily be a smooth curve. The horizontal axis for each ROC plot is labelled in terms of specificity decreasing from 1.0 to 0.0. This style of labelling is (1-specificity ranging from 0.0 to 1.0).

The position of the ROC curve depends on the degree of overlap of the distributions of the test measurement in diseased and non-diseased. Where a test clearly discriminates between diseased and non-diseased such that there is no or little overlap of distributions, the ROC curve will indicate that high sensitivity is achieved with a high specificity, that is the curve approaches the upper left hand corner of the graph where sensitivity is 1 and specificity is 1. If the distributions of test results in diseased and non-diseased coincide, the test would be completely uninformative and its ROC curve would be the upward diagonal of the square.

The ROC curves may be symmetrical about the sensitivity=specificity line (the downward diagonal of the square) or not symmetrical. Asymmetrical curves typically occur when the distribution of the test measurement in those with disease has more or less variability than the distribution in non-diseased people. Increased variability might occur, for example, where disease may cause a biomarker both to rise and become more erratic; reduced variability might occur where disease may lower biomarker values to a bounding level such as a lower level of detection.

COUPLED FOREST PLOTS

Forest plots for diagnostic test accuracy report the number of true positives and false negatives in diseased and true negatives and false positives in non-diseased participants in each study, and the estimated sensitivity and specificity, together with confidence intervals. The plots are known as coupled forest plots as they contain two graphical sections: one depicting sensitivity, and one specificity. The order of the studies can be sorted, often they are presented sorted by values of sensitivity, or grouped by test type or covariate values. Whilst it is possible to observe heterogeneity in sensitivity and specificity individually on such plots, it is not as easy to visualize whether there are threshold-like relationships. Summary statistics computed from meta-analyses can be added to coupled forest plots.

		PRISMA CHECHLIST<br>[Preferred Reporting Items for Systematic Reviews and Meta-Analyses:]	
**Section/topic**	**#**	**Checklist item**	**Reported on page #**
**TITLE**			
Title	1	Identify the report as a systematic review, meta-analysis, or both.	
**ABSTRACT**			
Structured summary	2	Provide a structured summary including, as applicable: background; objectives; data sources; study eligibility criteria, participants, and interventions; study appraisal and synthesis methods; results; limitations; conclusions and implications of key findings; systematic review registration number.	
**INTRODUCTION**			
Rationale	3	Describe the rationale for the review in the context of what is already known.	
Objectives	4	Provide an explicit statement of questions being addressed with reference to participants, interventions, comparisons, outcomes, and study design (PICOS).	
**METHODS**			
Protocol and registration	5	Indicate if a review protocol exists, if and where it can be accessed (e.g., Web address), and, if available, provide registration information including registration number.	
Eligibility criteria	6	Specify study characteristics (e.g., PICOS, length of follow-up) and report characteristics (e.g., years considered, language, publication status) used as criteria for eligibility, giving rationale.	
Information sources	7	Describe all information sources (e.g., databases with dates of coverage, contact with study authors to identify additional studies) in the search and date last searched.	
Search	8	Present full electronic search strategy for at least one database, including any limits used, such that it could be repeated.	
Study selection	9	State the process for selecting studies (i.e., screening, eligibility, included in systematic review, and, if applicable, included in the meta-analysis).	
Data collection process	10	Describe method of data extraction from reports (e.g., piloted forms, independently, in duplicate) and any processes for obtaining and confirming data from investigators.	
Data items	11	List and define all variables for which data were sought (e.g., PICOS, funding sources) and any assumptions and simplifications made.	

Risk of bias in individual studies	12	Describe methods used for assessing risk of bias of individual studies (including specification of whether this was done at the study or outcome level), and how this information is to be used in any data synthesis.	
Summary measures	13	State the principal summary measures (e.g., risk ratio, difference in means).	
Synthesis of results	14	Describe the methods of handling data and combining results of studies, if done, including measures of consistency (e.g., I^2) for each meta-analysis.	

Section/topic	#	Checklist item	Reported on page #
Risk of bias across studies	15	Specify any assessment of risk of bias that may affect the cumulative evidence (e.g., publication bias, selective reporting within studies).	
Additional analyses	16	Describe methods of additional analyses (e.g., sensitivity or subgroup analyses, meta-regression), if done, indicating which were pre-specified.	
**RESULTS**			
Study selection	17	Give numbers of studies screened, assessed for eligibility, and included in the review, with reasons for exclusions at each stage, ideally with a flow diagram.	
Study characteristics	18	For each study, present characteristics for which data were extracted (e.g., study size, PICOS, follow-up period) and provide the citations.	
Risk of bias within studies	19	Present data on risk of bias of each study and, if available, any outcome level assessment (see item 12).	
Results of individual studies	20	For all outcomes considered (benefits or harms), present, for each study: (a) simple summary data for each intervention group (b) effect estimates and confidence intervals, ideally with a forest plot.	
Synthesis of results	21	Present results of each meta-analysis done, including confidence intervals and measures of consistency.	
Risk of bias across studies	22	Present results of any assessment of risk of bias across studies (see Item 15).	
Additional analysis	23	Give results of additional analyses, if done (e.g., sensitivity or subgroup analyses, meta-regression [see Item 16]).	
**DISCUSSION**			
Summary of evidence	24	Summarize the main findings including the strength of evidence for each main outcome; consider their relevance to key groups (e.g., healthcare providers, users, and policy makers).	

Limitations	25	Discuss limitations at study and outcome level (e.g., risk of bias), and at review-level (e.g., incomplete retrieval of identified research, reporting bias).	
Conclusions	26	Provide a general interpretation of the results in the context of other evidence, and implications for future research.	
**FUNDING**			
Funding	27	Describe sources of funding for the systematic review and other support (e.g., supply of data); role of funders for the systematic review.	

From: Moher D, Liberati A, Tetzlaff J, Altman DG, The PRISMA Group (2009). Preferred Reporting Items for Systematic Reviews and Meta-Analyses: The PRISMA Statement. PLoS Med 6(6):
e1000097. doi:10.1371/journal.pmed1000097

For more information, visit: **www.prisma-statement.org**.

***Table 1.* Risk of Bias and Applicability Judgments in QUADAS-2**

Domain	Patient Selection	Index Test	Reference Test	Flow & timing
Description	Describe methods of patient selection<br><br>Describe included patients (previous testing, presentation, intended use of index test, and setting)	Describe the index test and how it was conducted and interpreted	Describe the reference standard and how it was conducted and interpreted	Describe any patients who did not receive the index tests or reference standard or who were excluded from the 2 x 2 table (refer to flow diagram)<br><br>Describe the interval and any interventions between index tests and the reference standard
Signaling questions (yes, no, or unclear)	Was a consecutive or random sample of patients enrolled?<br><br>Was a case–control design avoided?<br><br>Did the study avoid inappropriate exclusions?	Were the index test results interpreted without knowledge of the results of the reference standard?<br><br>If a threshold was used, was it prespecified?	Is the reference standard likely to correctly classify the target condition?<br><br>Were the reference standard results interpreted without knowledge of the results of the index test?	Was there an appropriate interval between index tests and reference standard?<br><br>Did all patients receive a reference standard?<br><br>Did all patients receive the same reference standard?<br>Were all patients included in the analysis?
Risk of bias (high, low, or unclear)	Could the selection of patients have introduced bias?	Could the conduct or interpretation of the index test have introduced bias?	Could the reference standard, its conduct, or its interpretation have introduced bias?	Could the patient flow have introduced bias?
Concerns about applicability (high, low, or unclear)	Are there concerns that the included patients do not match the review question?	Are there concerns that the index test, its conduct, or its interpretation differ from the review question?	Are there concerns that the target condition as defined by the reference standard does not match the review question?	

STARD checklist [PDF-file] [WORD version]

The STARD checklist consist of 25 items. Please, click on the description of the items for the rationale of the item and an example.

Section and Topic	Item		On page
TITLE/ABSTRACT/ KEYWORDS	1	Identify the article as a study of diagnostic accuracy(recommend MeSH heading 'sensitivity and specificity').	
INTRODUCTION	2	State the research questions or study aims, such as estimating diagnostic accuracy or comparing accuracy between tests or across participant groups.	
METHODS			
Participants	3	Describe the study population: The inclusion and exclusion criteria, setting and locations where the data were collected.	
	4	Describe participant recruitment: Was recruitment based on presenting symptoms, results from previous tests, or the fact that the participants had received the (evaluated) index tests or the (golden) reference standard?	
	5	Describe participant sampling: Was the study population a consecutive series of participants defined by the selection criteria in items 3 and 4? If not, specify how participants were further selected.	
	6	Describe data collection: Was data collection planned before the index test and reference standard were performed (prospective study) or after (retrospective study)?	
Test methods	7	Describe the reference standard and its rationale.	
	8	Describe technical specifications of material and methods involved including how and when measurements were taken, and/or cite references for index tests and reference standard.	

**Section and Topic**	**Item**		**On page**
	9	Describe definition of and rationale for the units, cut-offs and/or categories of the results of the index tests and the reference standard.	
	10	Describe the number, training and expertise of the persons executing and reading the index tests and the reference standard.	
	11	Describe whether or not the readers of the index tests and reference standard were blind (masked) to the results of the other test and describe any other clinical information available to the readers.	
Statistical methods	12	Describe methods for calculating or comparing measures of diagnostic accuracy, and the statistical methods used to quantify uncertainty (e.g. 95% confidence intervals).	
	13	Describe methods for calculating test reproducibility, if done.	
RESULTS			
Participants	14	Report when study was done, including beginning and ending dates of recruitment.	
	15	Report clinical and demographic characteristics of the study population (e.g. age, sex, spectrum of presenting symptoms, co morbidity, current treatments, recruitment centers).	
	16	Report the number of participants satisfying the criteria for inclusion that did or did not undergo the index tests and/or the reference standard; describe why participants failed to receive either test (a flow diagram is strongly recommended).	
Test results	17	Report time interval from the index tests to the reference standard, and any treatment administered between.	
	18	Report distribution of severity of disease (define criteria) in those with the target condition; other diagnoses in participants without the target condition.	

**Section and Topic**	**Item**		**On page**
	19	Report a cross tabulation of the results of the index tests (including indeterminate and missing results) by the results of the reference standard; for continuous results, the distribution of the test results by the results of the reference standard.	
	20	Report any adverse events from performing the index tests or the reference standard.	
Estimates	21	Report estimates of diagnostic accuracy and measures of statistical uncertainty (e.g. 95% confidence intervals).	
	22	Report how indeterminate results, missing responses and outliers of the index tests were handled.	
	23	Report estimates of variability of diagnostic accuracy between subgroups of participants, readers or centres, if done.	
	24	Report estimates of test reproducibility, if done.	
DISCUSSION	25	Discuss the clinical applicability of the study findings.	

REFERENCES

1. Shepard CW, Finelli L, Alter MJ. Global epidemiology of hepatitis C virus infection. Lancet Infect Dis. 2005;5:558-67.
2. Hanafiah KM, Groeger J, Flaxman AD, Wiersma ST. Global epidemiology of hepatitis virus C infection: New estimates of age specific antibody to HCV sero-prevalence. Hepatology 2013; 57:1330-1342.
3. Chen SL, Morgan TR. The natural history of hepatitis C virus (HCV) infection. Int J Med Sci. 2006;3:47-52.
4. Averhoff FM, Glass N, Holtzman D. Global burden of hepatitis C: Consideration for healthcare providers in the United States. Clin Infec Dis 2012;55 (Suppl 1):S10-15.
5. Alter MJ. Epidemiology of viral hepatitis and HIV co-infection. J Hepatol. 2006;44:S6-9.
6. Surveillance and Risk Assessment Division, Centre for Infectious Disease Prevention and Control, Public Health Agency of Canada. I-Track: Enhanced Surveillance of Risk Behaviours Among People who Inject Drugs. Phase I report, August 2006. Ottawa, Ontario, Canada: Public Health Agency of Canada, 2006.
7. Nyirenda M, Beadsworth MB, Stephany P, Hart CA, Hart IJ, Munthali C, et al. Prevalence of infection with hepatitis B and C virus and coinfection with HIV in medical inpatients in Malawi. J Infect. 2008;57:72-7.
8. Alter MJ, Kuhnert WL, Finelli L; Centers for Disease Control and prevention. Guidelines for laboratory testing and result reporting of antibody to hepatitis C virus. Center for Disease Control and Prevention. MMWR RECOMM REP. 2003; 52 (RR)-3):1-13, 15; Quiz CE 1 to 4.
9. John AS, Price CP. Economic Evidence and Point-of-Care Testing. Clin Biochem Rev 2013:34; 61-74.
10. Centers for Disease Control and Prevention, "Update: HIV counseling and testing using rapid tests—United States, 1995," Morbidity Mortality Weekly Report, vol. 47, pp. 211–215.
11. Mabey D, Peeling RW, Perkins MD. Rapid and simple point of care diagnostics for STIs (Editorial). Sex Transm Infect 2001; 77: 397-398.
12. Shivkumar S, Peeling R, Jafari Y, Joseph L, Pai NP. Accuracy of rapid and point of care screening tests for hepatitis C: A systematic review and meta-analysis. Ann Intern Med 2012; 157:558-566.
13. Higgins JP, Thompson SG. Quantifying heterogeneity in a meta-analysis. Stat Med 2002; 21:1539-1558.
14. Khuroo MS, Khuroo M, Farahat KLC, Kagevi I. Treatment with proton pump inhibitors in acute non-variceal upper gastrointestinal bleeding: A meta-analysis. J Gastroenterol Hepatol 2005; 20: 11–25.
15. Moher D, Liberati A, Tetzlaff J, Altman DG, The PRISMA Group (2009). Preferred Reporting Items for Systematic Reviews and Meta-Analyses: The PRISMA Statement. PLoS Med 2009; 6(7): e1000097.

16. Whiting PF, Rutjes AW, Westwood ME, Mallett S, Deeks JJ, Reitsma JB, et al; QUADAS-2 Group. QUADAS-2: a revised tool for the quality assessment of diagnostic accuracy studies. Ann Intern Med. 2011; 155(8):529-536.
17. Bossuyt PMM , Reitsma JB , Bruns DE, Gatsonis CA, Glasziou PP, Irwig LM et al. Standards for reporting of diagnostic accuracy. Toward complete and accurate reporting of studies of diagnostic accuracy: the STARD initiative. Ann Int Med 2003;138 :40–44.
18. Reistma JB, Glas AS, Rutjes AW, Scholten RJ, Bossuyt PM, Zwinderman AH. Bivariate analysis of sensitivity and specificity produces informative summary measures lin diagnostic reviews. J Clin Epidemiol 2005; 58:982-990.
19. DerSimonian, R. & Laird, N. Meta-analysis in clinical trials. Controlled Clinical Trials 1986; 7: 177–188.
20. Vamvakas EC. Applications of Meta-Analysis in Pathology Practice. Am J Clin Pathol 2001; 116 (Suppl 1):S47-S64.
21. Wallace CB, Schmid CH, Lau J & Trikalinos TA. Meta-Analyst: Software for meta-analysis of binary, continuous and diagnostic data. BMC Med Res Methodol 2009;9:80.
