# Supplementary material for: Diagnostic Accuracy of Point-of-Care Tests for Hepatitis C Virus Infection: A Systematic Review and Meta-Analysis
Source: PLoS One. 2015 Mar 27;10(3):e0121450. doi: 10.1371/journal.pone.0121450 (PMC4376712; doi:10.1371/journal.pone.0121450)
Supplement: S1 Table — (DOCX) [file pone.0121450.s009.docx]

| Table S1.  2×2 Data Table of Included studies evaluating 30 point-of-Care test with 73 data points. | | | | | | | | | | |
| --- | --- | --- | --- | --- | --- | --- | --- | --- | --- | --- |
| Study NO. | Data points | Author (reference) | Year | Index Test | Specimen | Sample Size¥ | True +VE | False -VE | False +VE | True -VE |
| 1 |  | Poovorawari^23^ | 1994 | Spot | Serum | 192 | 41 | 1 | 11 | 139 |
| 2 |  | Mvere^24^ | 1996 | Spot | Serum | 206 | 10 | 1 | 4 | 191 |
| 3 |  | Montebugnoli^25^ | 1999 | Rapid | Whole blood | 100 | 50 | 0 | 1 | 49 |
| 4 |  | Kaur^26^ | 2000 | BiDot | Serum | 2754 | 28 | 4 | 0 | 2722 |
| 5 |  | Buti^27^ | 2000 | Multiplo | Serum | 188 | 135 | 3 | 0 | 50 |
| 6 |  | Yuen^28^ | 2001 | SM-HCV | Serum | 290 | 98 | 3 | 0 | 189 |
| 7 |  | WHO-1^29^ | 2001 | Advanced | Serum | 257 | 66 | 2 | 10 | 179 |
| 7 |  | WHO-1^29^ | 2001 | TriDot | Serum | 257 | 68 | 0 | 39 | 150 |
| 7 |  | WHO-1^29^ | 2001 | Serodia | Serum | 257 | 68 | 0 | 1 | 188 |
| 7 |  | WHO-1^29^ | 2001 | Spot | Serum | 257 | 68 | 0 | 14 | 175 |
| 7 |  | WHO-1^29^ | 2001 | SeroCard | Serum | 257 | 67 | 1 | 0 | 189 |
| 8 |  | WHO-2^30^ | 2001 | TriDot 4^th^ | Serum | 257 | 68 | 0 | 5 | 184 |
| 8 |  | WHO-2^30^ | 2001 | Genedia | Serum | 257 | 67 | 1 | 3 | 186 |
| 9 |  | WHO-3^31^ | 2002 | SDBioline | Serum | 257 | 64 | 4 | 0 | 189 |
| 10 |  | Hui^32^ | 2002 | SM-HCV | Whole blood | 197 | 91 | 18 | 0 | 88 |
| 11 |  | Daniel^33^ | 2005 | TriDot | Serum | 2590 | 138 | 1 | 24 | 2427 |
| 12 |  | Scheiblauer^12^ | 2006 | Acon | Serum | 381 | 187 | 13 | 0 | 181 |
| 12 |  | Scheiblauer^12^ | 2006 | HepaScan | Serum | 381 | 183 | 17 | 0 | 181 |
| 12 |  | Scheiblauer^12^ | 2006 | TriDot | Serum | 381 | 199 | 1 | 0 | 181 |
| 12 |  | Scheiblauer^12^ | 2006 | Genedia | Serum | 381 | 193 | 7 | 1 | 180 |
| 12 |  | Scheiblauer^12^ | 2006 | i+Lab | Serum | 381 | 45 | 155 | 0 | 181 |
| 12 |  | Scheiblauer^12^ | 2006 | Dipstick | Serum | 381 | 190 | 10 | 0 | 181 |
| 12 |  | Scheiblauer^12^ | 2006 | Assure | Serum | 381 | 198 | 2 | 0 | 181 |
| 12 |  | Scheiblauer^12^ | 2006 | SPAN | Serum | 381 | 196 | 4 | 1 | 180 |
| 12 |  | Scheiblauer^12^ | 2006 | ImmunoRAPIDO | Serum | 381 | 199 | 1 | 2 | 179 |
| 13 |  | Njouom^34^ | 2006 | Hexagon | Plasma | 329 | 103 | 58 | 0 | 168 |
| 13 |  | Njouom^34^ | 2006 | Immunocomb | Plasma | 329 | 160 | 1 | 17 | 151 |
| 14 |  | Torane^35^ | 2008 | Spot | Whole blood | 60 | 0 | 30 | 0 | 30 |
| 15 |  | Nyirenda^36^ | 2008 | Spot | Serum | 202 | 2 | 7 | 7 | 186 |
| 16 |  | Ivantes^37^ | 2010 | Bioeasy | Whole blood | 71 | 30 | 0 | 3 | 38 |
| 17 |  | Lee-1^38^ | 2010 | OraQuick | Oral fluid | 572 | 122 | 1 | 0 | 449 |
| 17 |  | Lee-1^38^ | 2010 | OraQuick | Whole blood | 572 | 123 | 0 | 0 | 449 |
| 17 |  | Lee-1^38^ | 2010 | OraQuick | Finger stick | 572 | 123 | 0 | 0 | 449 |
| 17 |  | Lee-1^38^ | 2010 | OraQuick | Plasma | 572 | 123 | 0 | 1 | 448 |
| 17 |  | Lee-1^38^ | 2010 | OraQuick | Serum | 572 | 123 | 0 | 1 | 448 |
| 18 |  | Lee-2^39^ | 2011 | OraQuick | Oral fluid | 2176 | 739 | 14 | 5 | 1418 |
| 18 |  | Lee-2^39^ | 2011 | OraQuick | Whole blood | 2178 | 753 | 2 | 2 | 1421 |
| 18 |  | Lee-2^39^ | 2011 | OraQuick | Finger stick | 2176 | 752 | 2 | 1 | 1421 |
| 18 |  | Lee-2^39^ | 2011 | OraQuick | Plasma | 2178 | 755 | 1 | 2 | 1420 |
| 18 |  | Lee-2^39^ | 2011 | OraQuick | Serum | 2180 | 756 | 1 | 1 | 1422 |
| 19 |  | Smith-1^40^ | 2011 | Chembio | Serum | 1081 | 525 | 12 | 1 | 543 |
| 19 |  | Smith-1^40^ | 2011 | Multiplo | Serum | 1081 | 474 | 63 | 1 | 543 |
| 19 |  | Smith-1^40^ | 2011 | OraQuick | Serum | 1081 | 533 | 4 | 3 | 541 |
| 20 |  | Smith-2^41^ | 2011 | Chembio | Oral fluid NY | 197 | 136 | 16 | 10 | 35 |
| 20 |  | Smith-2^41^ | 2011 | Chembio | Oral fluid Denver | 282 | 178 | 17 | 3 | 84 |
| 20 |  | Smith-2^41^ | 2011 | Chembio | Blood Denver | 389 | 264 | 20 | 4 | 101 |
| 20 |  | Smith-2^41^ | 2011 | OraQuick | Oral fluid NY | 285 | 202 | 12 | 3 | 68 |
| 20 |  | Smith-2^41^ | 2011 | Multiplo | Whole blood | 432 | 302 | 82 | 9 | 39 |
| 20 |  | Smith-2^41^ | 2011 | OraQuick | Oral fluid Seattle | 265 | 177 | 19 | 0 | 69 |
| 20 |  | Smith-2^41^ | 2011 | OraQuick | Whole blood Seattle | 266 | 189 | 8 | 0 | 69 |
| 21 |  | Drobnik^42^ | 2011 | OraQuick | Oral fluid | 484 | 92 | 7 | 3 | 382 |
| 22 |  | Cha^43^ | 2012 | OraQuick | Oral fluid | 437 | 134 | 3 | 0 | 300 |
| 22 |  | Cha^43^ | 2012 | OraQuick | Serum | 400 | 200 | 0 | 0 | 200 |
| 23 |  | Maity^44^ | 2012 | TriDot | Serum | 300 | 120 | 6 | 0 | 174 |
| 23 |  | Maity^44^ | 2012 | SPAN | Serum | 300 | 132 | 0 | 0 | 168 |
| 23 |  | Maity^44^ | 2012 | SDBioline | Serum | 300 | 132 | 0 | 0 | 168 |
| 24 |  | Jewett^45^ | 2012 | Chembio | Blood | 406 | 103 | 8 | 5 | 290 |
| 24 |  | Jewett^45^ | 2012 | Chembio | Oral fluid | 406 | 90 | 20 | 7 | 288 |
| 24 |  | Jewett^45^ | 2012 | Multiplo | Blood | 409 | 82 | 32 | 26 | 269 |
| 25 |  | Kant^46^ | 2012 | Onsite | Serum | 185 | 82 | 1 | 12 | 90 |
| 26 |  | Kim^47^ | 2013 | SDBioline | Serum | 100 | 52 | 14 | 0 | 34 |
| 26 |  | Kim^47^ | 2013 | Genedia | Serum | 100 | 46 | 20 | 0 | 34 |
| 27 |  | Al-Tahish^48^ | 2013 | One-step | Serum | 100 | 49 | 1 | 0 | 50 |
| 27 |  | Al-Tahish^48^ | 2013 | TriDot 4^th^ | Serum | 100 | 49 | 1 | 0 | 50 |
| 27 |  | Al-Tahish^48^ | 2013 | Immunocomb | Serum | 100 | 48 | 2 | 0 | 50 |
| 28 |  | da Rosa^49^ | 2013 | Bioeasy | Serum | 307 | 100 | 3 | 0 | 204 |
| 28 |  | da Rosa^49^ | 2013 | ImmunoRAPIDO | Serum | 307 | 100 | 3 | 0 | 204 |
| 29 |  | OConnell^50^ | 2013 | OraQuick | Plasma | 674 | 333 | 2 | 1 | 338 |
| 29 |  | OConnell^50^ | 2013 | Instant | Plasma | 674 | 321 | 14 | 3 | 336 |
| 29 |  | OConnell^50^ | 2013 | Axiom | Plasma | 674 | 326 | 9 | 10 | 329 |
| 29 |  | OConnell^50^ | 2013 | CORE | Plasma | 674 | 323 | 12 | 7 | 332 |
| 29 |  | OConnell^50^ | 2013 | FirstVue | Plasma | 674 | 312 | 23 | 3 | 336 |
| 30 |  | Tagny^51^ | 2014 | Hexagon | Serum | 1998 | 26 | 15 | 28 | 1929 |
| ¥= All inconclusive index test results were clubbed with either false negative results (anti-HCV positive sera with inconclusive index test results) or false positive results (anti-HCV negative sera with inconclusive index test results).^13^ | | | | | | | | | | |
