# Supplementary material for: Diagnostic Accuracy of Point-of-Care Tests for Hepatitis C Virus Infection: A Systematic Review and Meta-Analysis
Source: PLoS One. 2015 Mar 27;10(3):e0121450. doi: 10.1371/journal.pone.0121450 (PMC4376712; doi:10.1371/journal.pone.0121450)
Supplement: S2 Table — (DOCX) [file pone.0121450.s010.docx]

| Table S2. List of 20 full-text artilces which were excluded from final analysis. | | |
| --- | --- | --- |
| Serial | **Full text article** | **Reason for exclusion** |
|  | Kamili S, Drobeniuc J, Araujo AC, Hayden TM. Laboratory Diagnostics for Hepatitis C Virus Infection. Clinical Infectious Diseases 2012;55(S1):S43–8 | Guidelines for HCV diagnosis. |
|  | Smith BD, Jewett A, Drrrobeniuc J, Kamili S. Rapid diagnostic HCV antibody assays. Antiviral Therapy 2012;17: 1409-13. | Guidelines for HCV diagnosis. |
|  | Smith BD, Drobeniuc J, Jewett A, et al. Evaluation of Three Rapid Screening Assays for Detection of Antibodies to Hepatitis C Virus. Journal Infectious Diseases 2011;204:825–31 | Guidelines for HCV diagnosis. |
|  | Wichroski MJ, Fang J, Eggers BJ, et al. High Throughput Screening and Rapid Inhibitor Triage Using an Infectious Chimeric Hepatitis C Virus. PLoS ONE 2012; 7(8): e42609. doi:10.1371/journal.pone.0042609 | Guidelines for HCV diagnosis. |
|  | Zoulim F. New nucleic acid diagnostic tests in viral hepatitis. Semin Liver Dis. 2006 ;26(4):309-17. | Guidelines for HCV diagnosis. |
|  | Cock LD*,*, Hutseb V, Verhaegena E, Quoilinb S, Vandenbergheb H, Vranckxa R. Detection of HCV antibodies in oral fluid. Journal Virological Methods 2004:122; 179–183. | Oral fluid testing. |
|  | Kania D, Bekal AM, Nagot N, et al. Combining rapid diagnostic tests and dried blood spot assays for point-of-care testing of human immunodeficiency virus, hepatitis B and hepatitis C infections in Burkina Faso, West Africa. Clin Microbiol Infect 2013; 19: E533–E541. | Dried blood spot testing. |
|  | Valcavi P , Medici MC, Casula F, Arcangeletti MC, De Conto F, Pinardi F, Calderaro A, Chezzi C, Dettori G. Evaluation of a total hepatitis C virus (HCV) core antigen assay for the detection of antigenaemia in anti-HCV positive individuals. J Med Virol 2004;73(3):397-403. | Development strategy for new HCV test. |
|  | Kargar M, Askari A, Doosti A, Ghorbani-Dalini S. Loop-Mediated Isothermal Amplification Assay for Rapid Detection of Hepatitis C virus. Indian J. Virol. 2012;23(1):18–23. | Development strategy for new HCV test. |
|  | Miedouge M , Legrand-Abravanel F, Lalanne C, Saune K, Izopet J. Laboratory evaluation of the UniCel DxI 800 analyser (Beckman Coulter) for detecting HBV and HCV serological markers. Clin Virol. 2009;44(2):134-7. | Development strategy for new HCV test. |
|  | Mohammadi-Yeganeh S, Paryan M, Mirab Samiee S, Kia V, Rezvan H. Molecular beacon probes–base multiplex NASBA Real-time for detection of HIV-1 and HCV. Iranian Journal Microbiology. 2012: *4(2); 47-54.* | Development strategy for new HCV test. |
| 1. . | Paryan M, Mohammadi-Yeganeh S, Mirab Samiee S, Rezvan H. Design and Development of a Multiplex Real-Time PCR Assay for Detection of HIV-1 and HCV Using Molecular Beacons. Indian J Microbiol 2012;52(3):456–463. DOI 10.1007/s12088-012-0271-1. | Development strategy for new HCV test. |
|  | van Doorn LJ , van Belkum A, Maertens G, Quint W, Kos T, Schellekens H. Hepatitis C virus antibody detection by a line immunoassay and (near) full length genomic RNA detection by a new RNA-capture polymerase chain reaction. J Med Virol. 1992;38(4):298-304. | Development strategy for new HCV test. |
|  | Wang Q-q, Zhang J, Hu J-s, et al. Rapid detection of hepatitis C virus RNA bya reverse transcription loop-mediated isothermal amplication assay. FEMS 2011. DOI:10.1111/j.1574-695X.2011.00828.x | Development strategy for new HCV test. |
|  | Batool A, Khan MI, Bano KA. Efficacy of immunoassay chromatography test for hepatitis-C antibodies detection. J Ayub Med Coll Abbottabad. 2009: 21(3):38-9. | Reference test for Index test positive sera only. |
|  | Desbois D , Vaghefi P, Savary J, Dussaix E, Roque-Afonso AM. Sensitivity of a rapid immuno-chromatographic test for hepatitis C antibodies detection. J Clin Virol 2008:41(2):129-33. | Index test on HCV positive sera only. |
|  | Firdaus R, Saha K, Sadhukhan PC. Rapid immunoassay alone is insufficient for the detection of hepatitis C virus infection among high-risk population. J Viral Hepat. 2013 Apr;20(4):290-3. | Reference test for Index test negative sera only. |
|  | Shahzamani K, Sabahi F, Merat S, et al. Rapid Low-cost Detection of Hepatitis C Virus RNA in HCVinfected Patients by Real-time RT-PCR using SYBR Green. Archives Iranian Medicine, 2011:14 (6); 396-400. | Index test for HCV positive sera only. |
|  | Constantine NT, Holm-Hansen C, Skaug N, Vasilescu F. Successful use of two rapid HCV assays in a high prevalence Romanian population. J Clin Lab Anal. 1994; 8(5):332-4. | Index test for HCV positive sera only. |
|  | Kwenti TE, Njouom R, Njunda LA, Kamga HLF. Comparison of an Immunochromatographic Rapid Strip Test, ELISA and PCR in the Diagnosis of Hepatitis C in HIV Patients in Hospital Settings in Cameroon. Clinical Medicine and Diagnostics. 2011; 1(1): 21-27. | Reference test for Index test positive sera only. |
