# Supplementary material for: Diagnostic Accuracy of Point-of-Care Tests for Hepatitis C Virus Infection: A Systematic Review and Meta-Analysis
Source: PLoS One. 2015 Mar 27;10(3):e0121450. doi: 10.1371/journal.pone.0121450 (PMC4376712; doi:10.1371/journal.pone.0121450)
Supplement: S3 Table — (DOCX) [file pone.0121450.s011.docx]

| **Table S3: Performance of Index Tests in relation to HCV Genotype Diversity** | | | | | | | |
| --- | --- | --- | --- | --- | --- | --- | --- |
| Study (reference) | Sera Panel | | | | Reference Test | Index Test | Results |
|  | Name | Number | | HCV Genotypes (G) |  |  |  |
| WHO-1 2001^29^ | World-wide performance panel | 20 | | G1-7; G 2-4; G 3-2; G4-3; non-typable-2; a; negative-2. | Five ELISA assays | Tridot, Advanced, Serodia, Spot, Serocard | 3 discordant results |
| WHO-2 2001^30^ | World-wide performance panel | 20 | | G1-7;G 2-4;G 3-2; G4-3; ; non-typable-2; a ; negative-2 | Five ELISA assays | Genedia, Tridot 4^th^ | No discordant results |
| Daniel et al 2005^33^ | - | | 60 | G1-16; G2-1; G3-31; G4-9; G6-3. | EIA (UBI, HCV EIA 4.0),  MEIA (Axsym Abbott lab., III). | Tridot | Index test failed to detect 4 sera  (G1-3 & G3b-one). |
| Scheiblauer et al 2006^12^ | ICBS master panel | | 200 | G1 to 6. | Five reference assays certified in the EU | Acon, HepaScan, TriDot, Serodia, Genedia Rapid, Genedia LF, i+Lab, Dipstick, Assure, Immunodot, ImmunoRAPIDO | False negative results were not linked to genotypes of the panel. |
