# Supplementary material for: Diagnostic Accuracy of Point-of-Care Tests for Hepatitis C Virus Infection: A Systematic Review and Meta-Analysis
Source: PLoS One. 2015 Mar 27;10(3):e0121450. doi: 10.1371/journal.pone.0121450 (PMC4376712; doi:10.1371/journal.pone.0121450)
Supplement: S4 Table — (DOCX) [file pone.0121450.s012.docx]

| **Table S4: Performance of Index tests for anti-HCV in relation to Cross-reactive HIV sera, Oral pathologies and conditions, Biological substances and storage and testing conditions.** | | | | | |
| --- | --- | --- | --- | --- | --- |
| **Study Variable** | **Study** | **Sample characteristics** | **Reference test** | **Index test** | **Results** |
| Cross reactive HIV sera | Nyirenda 2008 ^36^ | 226 sera (HIV positive-172; HIV/HBV co-infection 31; HIV/HCV co-infection-8 and triple infection-2). | Melisa HCV ag/ab ultra MEIA (Bio-Rad, Marnesla-Coquette France) | Spot test | True positive 2/9; False negative-7/193 (sensitivity 22%, specificity 96%) |
|  | Smith-1 2011^40^ | 1100 high-risk sera (IV drug users);  HIV positive 43, HCV positive 26. | Ortho VITROS anti-HCV chemiluminescent immunometric assay | Chembio, Multiplo, OraQuick | Significant false anti-HCV results with HIV positivity for the Chembio (9.3%) and Multiplo (16.3%) and not for OraQuick (2.3%). |
|  | Smith-2 2011^41^ | 1592 high risk sera (IV drug users); 99 were positive for HIV. | Ortho HCV version 3.0 ELISA | Chembio (oral & blood); Multiplo (Blood); OraQuick (Oral & blood). | False negative HCV results were associated with HIV positivity for only Chembio oral assay (9.1%) and not with other assays. |
| Oral pathology/ conditions | Lee-2 2011^39^ | 50 oral fluid samples (anti-HCV negative) from individuals with gingivitis, dentures, tobacco, food and drink, and oral care products with oral fluid from HCV positive and negative subjects. | EIA [Abbott AxSYM HCV version 3.0, Abbott La. USA]. | OraQuick | No interference with HCV positive and negative specimens observed. |
| Biological substances | Cha 2012^43^ | 30 HCV positive and 30 HCV negative sera mixed with bilirubin (10 mg/dl), hemoglobin (0.5 g/dl), lipid (300 mg/dl), rheumatoid factor, multipara and several viral infections were tested. | EIA | OraQuick | None of the interfering substances affected positivity of anti-HCV by OraQuick rapid test. |
| Storage & testing conditions | O’Connell 2013^50^ | 84 HCV positive and 84 HCV negative plasma were added to pathogen free whole blood to create 1:8 dilution. These samples underwent 4 storage conditions namely Normal, Hot storage; Hot testing and cold storage. | ELISA 3^rd^ generation | OraQuick, Instant view, Axiom, CORE, FirstVue | OraQuick had no significant effect on test performance by hot storage or hot testing or cold storage. Other 4 tests had significant decrease in sensitivity (not specificity) by storage and testing conditions. |
