# Supplementary material for: Diagnostic Accuracy of Point-of-Care Tests for Hepatitis C Virus Infection: A Systematic Review and Meta-Analysis
Source: PLoS One. 2015 Mar 27;10(3):e0121450. doi: 10.1371/journal.pone.0121450 (PMC4376712; doi:10.1371/journal.pone.0121450)
Supplement: S5 Table — (DOCX) [file pone.0121450.s013.docx]

| **Table S5: Performance of Index test in Seroconversion Panels** | | | | | |
| --- | --- | --- | --- | --- | --- |
| **Study** | **Panel µ** | **Reference** | **Index test** | **Antibody Detection µ** | **Performance µ** |
| WHO-1 2001^29^ | 39 sera from 4 commercial [Boston Biomedica Inc.-BBI] sera panels | Mono-Lisa anti-HCV plus (Biorad Pasteur); Ortho HCV 3 enhanced SAVe short incubation (Ortho Diagnostics) | TriDot | +2 | Consistent |
|  |  |  | Advanced | +0.25 | Consistent |
|  |  |  | Serodia | +0.2.5 | Missed one of the 4 panels |
|  |  |  | Spot | +2.5 | Consistent |
|  |  |  | Serocard | +6.5 | Missed one of the 4 panels |
| WHO-2 2001^30^ | 39 sera from 4 commercial [Boston Biomedica Inc.-BBI] sera panels | Mono-Lisa anti-HCV plus (Biorad Pasteur); Ortho HCV 3 enhanced SAVe short incubation (Ortho Diagnostics) | Genedia | +8 | Missed one of the 4 panels |
|  |  |  | TriDot 4^th^ | +9.7 | Missed one of the 4 panels |
| Scheiblauer et al 2006^12^ | 3 selected seroconversion panels [Boston Biomedica Inc.-BBI & ZeptoMetrix Corp, MA]. | Five reference assays certified in the EU [Abbott Architect; Abbott third Generation anti-HCV; Advia Centaur; Ortho HCV version 3.0; Vitro HCV]. | Acon | +16.67 | Consistent |
|  |  |  | Hepascan | +14.67 | Consistent |
|  |  |  | TriDot | +26.0 | Consistent |
|  |  |  | Serodia | +28.0 | Consistent |
|  |  |  | Genedia Rapid | +20.67 | Consistent |
|  |  |  | Genedia LF | +22.67 | Consistent |
|  |  |  | I+Lab | +28.0 | Consistent |
|  |  |  | Dipstick | +14.67 | Consistent |
|  |  |  | Assure | +28.0 | Consistent |
|  |  |  | Spot | +23.67 | Consistent |
|  |  |  | ImmunoRAPIDO | +14.67 |  |
| Lee-2010^38^ | Panel of 19 seroconversion sera panels | HCV EIA test | OraQuick | - 4.9 (95% CI 1.4-8.3) | Highly consistent |
| Lee-2011^39^ | Panel of 27 seroconversion sera panels | HCV EIA (Abbott AxSYM HCV VERSION 3.0). | OraQuick | -0.6 (95% CI 0.1-1.4) | Consistent |
| Cha 2013^43^ | Commercially available Seroconversion panel [SeraCare Life Sciences, MA] & reference panel from Korea Food and drug administration [KFDA]. | Five reference assays [Architect-Abbott; AxSYM-Abbott; E170-Roche; Advia Centaur-Siemens & Elecsys-Roche]. | OraQuick | -10 | Consistent; better than Asam, SDBioline and Green Cross |
| Kim et al 2013^47^ | 39 sera from 4 commercial [Boston Biomedica Inc.-BBI] sera panels | Advia Centaur Anti-HCV assay-Siemens | SDBioline | 0 | Consistent |
|  |  |  | Genedia | 0 | Poor; missed 2 panels with genotype 2 |
| O’Connell et al 2013^50^ | Five seroconversion panels (Sera-Care Life Sciences, Inc.) | Ortho HCV version 3 ELISA test [Ortho Clinical Diagnostics Raritan NJ]. | OraQuick | -3.6 | Highly consistent |
|  |  |  | CORE | +33.6 | Poor; missed 1 of the 5 panels and inconsistent in another 2 panels |
|  |  |  | Instant View | +20.2 | Poor; missed one of the 5 panels and inconsistent in one panel |
|  |  |  | Axiom | 0 | Poor; inconsistent in 2 panels |
|  |  |  | FirstVue | - | Poor; missed 4 of the 5 panels and inconsistent in fifth panel |
| µ=Seroconversion panels, each containing several samples taken at different time intervals early in the infection period (window period), were tested with index test kits. The results obtained were compared to those of the combined outcome of the reference tests; the difference in days of the first sample of a panel to become positive with index test as compared to the first positive result with the reference tests. If a test gave a positive result earlier than the reference tests the number of days difference in detection were rated as negative; if the test became positive later the number of days were rated as positive. The mean of the difference in time period for index test to become positive as compared to the reference tests was calculated. | | | | | |
